# Extraction of sugarcane bagasse arabinoxylan, integrated with enzymatic production of xylo-oligosaccharides and separation of cellulose

**DOI:** 10.1186/s13068-021-01993-z

**Published:** 2021-07-03

**Authors:** Leila Khaleghipour, Javier A. Linares-Pastén, Hamid Rashedi, Seyed Omid Ranaei Siadat, Andrius Jasilionis, Said Al-Hamimi, Roya R. R. Sardari, Eva Nordberg Karlsson

**Affiliations:** 1 Division Biotechnology, Department of Chemistry, Lund University, P. O. Box 124, 22100 Lund, Sweden; 2 Biotechnology Group, School of Chemical Engineering, College of Engineering, University of Tehran, Tehran, Iran; 3 Protein Research Center, ShahidBeheshti University, G. C., Tehran, Iran; 4 Center for Analysis and Synthesis, Department of Chemistry, Lund University, P. O. Box 124, 22100 Lund, Sweden

**Keywords:** Sugarcane bagasse, Xylan, Alkali extraction, Enzymatic hydrolysis, Xylo-oligosaccharides, Thermostable xylanases

## Abstract

Sugarcane processing roughly generates 54 million tonnes sugarcane bagasse (SCB)/year, making SCB an important material for upgrading to value-added molecules. In this study, an integrated scheme was developed for separating xylan, lignin and cellulose, followed by production of xylo-oligosaccharides (XOS) from SCB. Xylan extraction conditions were screened in: (1) single extractions in NaOH (0.25, 0.5, or 1 M), 121 °C (1 bar), 30 and 60 min; (2) 3 × repeated extraction cycles in NaOH (1 or 2 M), 121 °C (1 bar), 30 and 60 min or (3) pressurized liquid extractions (PLE), 100 bar, at low alkalinity (0–0.1 M NaOH) in the time and temperature range 10–30 min and 50–150 °C. Higher concentration of alkali (2 M NaOH) increased the xylan yield and resulted in higher apparent molecular weight of the xylan polymer (212 kDa using 1 and 2 M NaOH, vs 47 kDa using 0.5 M NaOH), but decreased the substituent sugar content. Repeated extraction at 2 M NaOH, 121 °C, 60 min solubilized both xylan (85.6% of the SCB xylan), and lignin (84.1% of the lignin), and left cellulose of high purity (95.8%) in the residuals. Solubilized xylan was separated from lignin by precipitation, and a polymer with β-1,4-linked xylose backbone substituted by arabinose and glucuronic acids was confirmed by FT-IR and monosaccharide analysis. XOS yield in subsequent hydrolysis by endo-xylanases (from glycoside hydrolase family 10 or 11) was dependent on extraction conditions, and was highest using xylan extracted by 0.5 M NaOH, (42.3%, using Xyn10A from *Bacillus halodurans*)*,* with xylobiose and xylotriose as main products. The present study shows successful separation of SCB xylan, lignin, and cellulose. High concentration of alkali, resulted in xylan with lower degree of substitution (especially reduced arabinosylation), while high pressure (using PLE), released more lignin than xylan. Enzymatic hydrolysis was more efficient using xylan extracted at lower alkaline strength and less efficient using xylan obtained by PLE and 2 M NaOH, which may be a consequence of polymer aggregation, via remaining lignin interactions.

**Supplementary Information:**

The online version contains supplementary material available at 10.1186/s13068-021-01993-z.

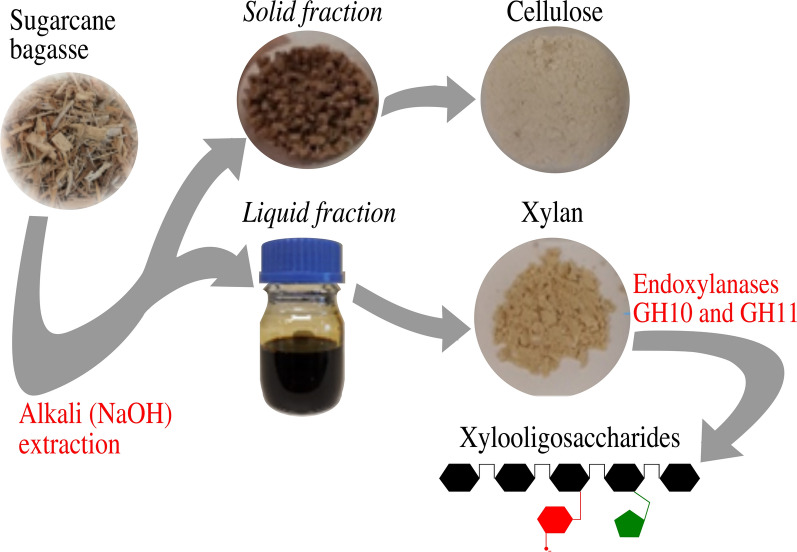

## Background

Lignocellulosic biomass is a cheap, abundant and renewable raw material and hence promising for sustainable production of biofuel [1], bioenergy and several value-added biomolecules, e.g., antioxidants [2], prebiotic xylo-oligosaccharides (XOS) [3], adhesives [4] and biodegradable plastics [5], in a biorefinery context. Sugarcane bagasse (SCB) is an important lignocellulosic residue that is generated in large amounts by sugar and alcohol industries. Processing of sugarcane, generates approximately 250–280 kg of SCB per ton processed sugarcane, which roughly yields 54 million tons of SCB annually throughout the world [6, 7]. Most of the SCB is burned in sugar mills for energy generation [8, 9].

SCB consists of 14–30% lignin, 35–50% cellulose and 22–36% hemicelluloses with limited amounts of extractives and ash [10–12]. The chemical composition, with high carbohydrate content and low ash content, makes SCB a suitable raw material for manufacturing of high value-products [11]. By fractionation of SCB into core chemical components (e.g., cellulose, hemicelluloses and lignin), each individual component can be used for the development of a variety of products, as demonstrated for other types of lignocellulosic byproducts [13]. The major hemicellulose component of SCB is xylose, showing that the major part of the hemicelluloses in bagasse are xylans. Xylans consists of a backbone of xylose residues that are linked via β-1-4 glycosidic bonds, substituted to varying degrees with, e.g., arabinose, glucuronic acid, 4-O-methyl and acetyl esters. The type and degree of branching are determined by the xylan source, resulting in sub-categories of xylans; including homoxylan, arabinoxylan, glucuronoxylan and arabinoglucuronoxylans [14].

Hemicelluloses have a very wide range of application. They can, dependent on the type of hemicellulose, be hydrolyzed into the monosaccharides pentoses (xylose and arabinose) or hexoses (glucose, galactose and mannose) and then be further converted in processes for production of bioethanol and other value-added chemicals (which for xylans include 5-hydroxymethylfurfural (HMF), furfural, levulinic acid and xylitol) [15]. Biopolymers, based on hemicellulose, also have use as viscosity modifiers in food packaging film, as wet strength additives in papermaking and as tablet binders [16]. Moreover, the xylan types of hemicelluloses have lately gained interest for the development of protocols for XOS production. XOS have applications as food ingredients with beneficial health effects [17], and are value-added products from the hemicellulosic xylan fraction that can be used as functional foods and prebiotics [3]. XOS are composed of xylose residues with a degree of polymerization (DP) from 2 to 10 xylose units [18] although up to 20 units are still considered as XOS [19]. XOS are classified as non-digestible oligosaccharides and considered prebiotic compounds, which stimulate the growth of probiotic bacteria such as *Bifidobacterium* and *Lactobacillus* [20] that maintain gastrointestinal health in human beings. Moreover, they are regarded as safe for use by both FDA and EFSA [21]. A number of studies indicate beneficial health effects of XOS, like reduction of risk for colon cancer, prevention of cardiovascular diseases and diabetes, increased absorption of nutrients, fortification of the immune system, anti-inflammatory and anticariogenic effects [12, 22–26]. Because of their multidimensional properties and wide application range, they have gained attention from food, feed, pharmaceutical and recently cosmetic industries [19, 27]. The worldwide market for XOS is expected to grow rapidly, estimated to reach 130 million US$ in 2023, from 93 million US$ in 2017, according to a new GIR (Global Info Research) study [28] in *The market reports* (2018).

Cellulose is a polysaccharide consisting of a linear chain of several hundreds to many thousands of β-1,4-linked D-glucose units. It is the most abundant biopolymer in nature and is therefore attractive as a sustainable raw material for industrial processes [29]. Many properties of cellulose depend on its degree of polymerization, which varies with origin. The main application of cellulose is in manufacturing paper and paperboard, but it can for example also be hydrolyzed into glucose and used for production of bioethanol [11]. Both cellulose and hemicellulose (including the various types of xylans) can, as stated above, also be used for other applications, such as the production of films, composites, nanostructures and new materials [30–34]. Therefore, the biorefinery perspective of SCB as investigated here, is an interesting alternative that can act as driving force towards separating and utilizing the polymers from this type of industrial residue.

Various types of “pretreatment” processes are essential to facilitate separation of core chemical components of lignocellulosic biomass, due to the structural robustness of plant cell walls. This recalcitrance is due to the crosslinking between the polysaccharides (cellulose and hemicellulose) and the lignin via ester and ether linkages [35]. In addition, hydrogen bond networks between cellulose and hemicelluloses may hinder liberation of the hemicellulose [36]. Pretreatment processes are often followed by enzymatic (or acid) hydrolysis to reduce the size of the polysaccharides, that are subsequently released in oligosaccharide or monosaccharide forms for further conversion by microorganisms.

A variety of effective pretreatment methods have been investigated to solubilize and fractionate components including dilute acid pretreatment [37], liquid hot water extraction [38], steam explosion [39], ionic liquids and alkaline extraction. The choice of extraction method depends on the purpose and the final application, and whether or not polysaccharide forms are desirable. Hence, dependent on the purpose, both alkali or acid pretreatment can be applied successfully. Alkali pretreatment disrupts the cell wall of lignocellulosic materials by dissolving hemicellulose and lignin, and decrease the crystallinity of cellulose [40], leading to final products in polysaccharide forms. Acid pretreatment results in high monosaccharide yield, accompanied with formation of byproducts [41]. The alkali pretreatment can be advantageous compared to acid pretreatment as less degradation of sugars occurs [12, 42] and better effectiveness in hemicellulose solubilization are reported [43, 44]. The alkali pretreatment has also been proposed as promising to attain complete utilization of lignocelluloses without impact to the environment [45].

The remaining insoluble residue is mainly expected to be cellulose that can be used for different applications. Removal of hemicellulose (as well as lignin) from this cellulose fraction can improve accessibility of hydrolytic glucan-degrading enzymes, resulting in more efficient degradation to monosaccharides, promoting a more efficient conversion to ethanol in subsequent yeast fermentations, facilitating an economically feasible production process. Moreover, by removal of hemicellulose and lignin, the formation of inhibitory degradation products in the subsequent yeast fermentation is avoided [16]. When hemicellulose extraction is performed with alkaline methods, the residual glucan-enriched insoluble material seems suitable for glucose production employing low cellulase loading [46]. Therefore, integrated bio-refining with separation of cellulose, hemicellulose and lignin, allows production of various value-added products (such as ethanol, sugar-based polyesters, and other chemicals and biopolymers) and offers a tremendous value-adding opportunity [16, 44].

The present project aims to develop an integrated scheme for the extraction of xylan and cellulose, separation of xylan and lignin, followed by enzymatic conversion of xylan into XOS. The application of alkali combined with autoclaving enables maximal recovery of hemicellulosic xylan from SCB, separating it from the cellulose fraction. The extracted xylan is, after separation from the lignin stream, enzymatically hydrolyzed, using different endo-acting xylanases to produce XOS, thus verifying the possibility to enzymatically convert the purified xylan fraction. To date, a wide number of raw materials are tested for XOS production including barley hulls, corn cobs [47], corn husk, almond shell, rice hulls, tobacco stalks, rye straw, cotton stalks, etc. [48]. Here, we explore SCB xylanase raw material for XOS production, showing the importance of both the choice of enzyme and choice of fractionation process for further conversion of the material.

## Results and discussion

**Fig. 1:**
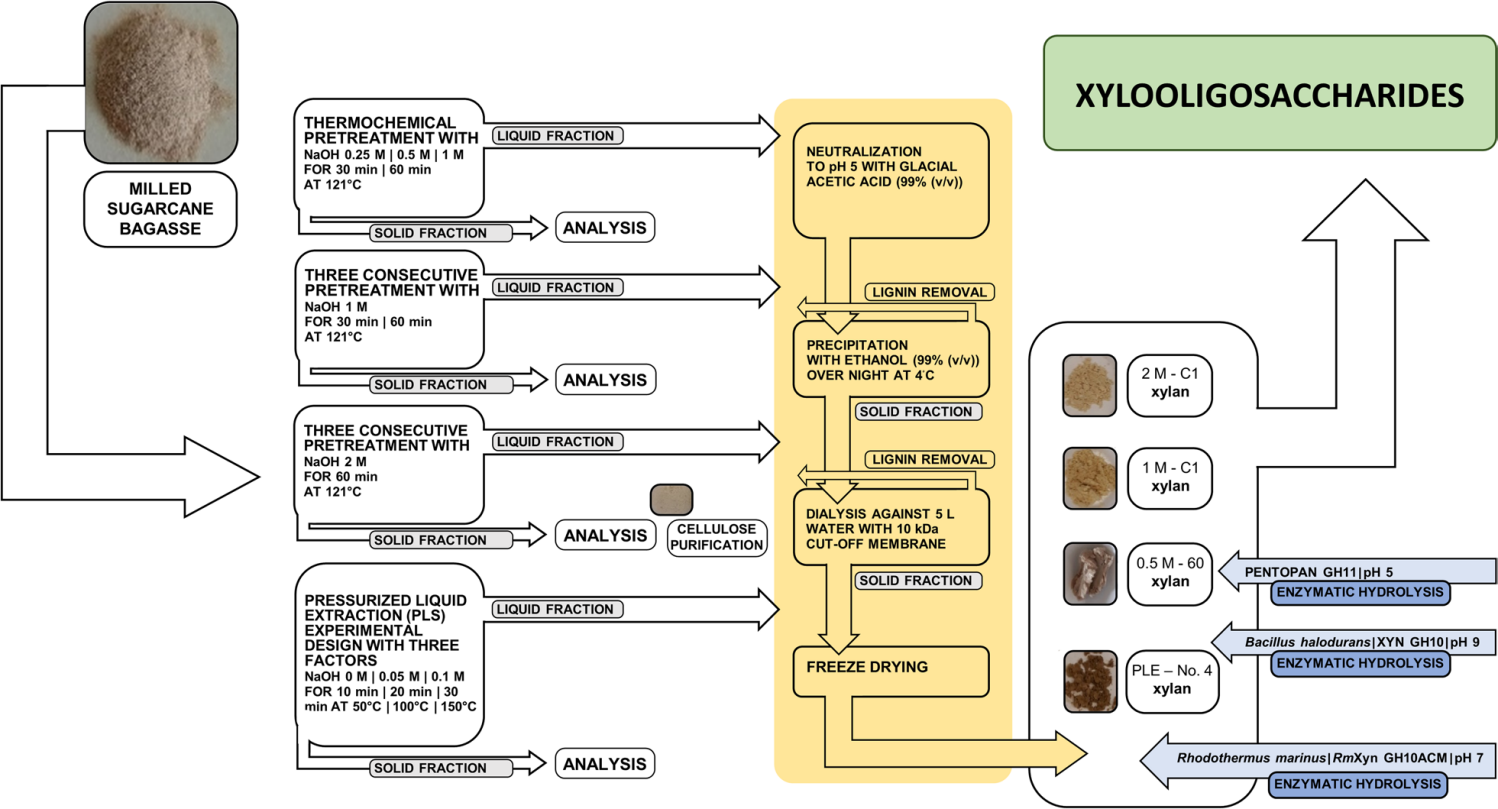
Process scheme for the extraction and purification of xylan, cellulose and xylo-oligosaccharides (XOS)

Sugarcane bagasse (SCB) is an underutilized byproduct, rich in cellulose and hemicellulose (mainly xylan) with limited amounts of extractives and ash, and is hence suitable for upgrading. Therefore, the present work aims to develop an integrated scheme for the separation of xylan, lignin and cellulose, to upgrade the raw material. The separation is followed by enzymatic conversion of xylan into XOS, which to date has been made to limited extent from this material [49, 50]. The used methods combine physico-chemical treatments (pressure, alkali and temperature) for xylan extraction, precipitation allowing separation of xylan from lignin, and acidic cleaning of the cellulose from the solid residuals (Fig. 1).

### Chemical characterization of the sugarcane bagasse

The first criterion for product valorization from lignocellulosic biomass is the suitability of the raw material, with high hemicellulose and cellulose content vis-a-vis its overall composition. SCB is previously reported to consist of approximately 70% carbohydrates [10, 11].The overall composition of the SCB-batch used here (Table 1) and the distribution of neutral monosaccharides (Table 2) shows that the results are in agreement with the composition reported by other groups [12, 51]. The total carbohydrate content of the batch was 67.3% of the dry weight (including uronic acids, Table 1), with the predominant components being glucose (36.6%, assumed to originate from cellulose) and xylose (25.7%, from the hemicellulose xylan) (Table 2). The resulting xylose:arabinose ratio was 11:1, and the xylose:glucuronic acid ratio was 14:1. This relatively low degree of substitution indicates good potential for endoxylanases to hydrolyze non-substituted parts of the xylan backbone into XOS.

**Table 1:** Chemical composition of raw sugarcane bagasse (% of dry weight)

Moisture	Ash	Protein	Uronic acid	Acid-insoluble lignin (Klasson)	Acid-soluble lignin	Total lignin	Total (neutral) carbohydrates
3.66 ± 0.03	3.92 ± 0.07	1.52 ± 0.29	1.87 ± 0.14	15.19 ± 0.42	4.91 ± 0.26	20.10 ± 0.68	65.42 ± 0.88

**Table 2:** Monosaccharide composition of structural carbohydrates in raw sugarcane bagasse (% on dry basis), and recalculation to polysaccharides

Arabinose	Galactose	Glucose	Xylose	Mannose	Cellulose	Hemicellulose
2.24 ± 0.07	0.72 ± 0.0	36.58 ± 0.13	25.71 ± 0.67	0.16 ± 0.05	32.97 ± 0.11	25.39 ± 0.66

The column cellulose represents a recalculation of the glucose content, using the correction factor 0.9 (based on the NREL procedure). The column hemicellulose represents a recalculation of the xylose, arabinose, galactose and mannose, using the correction factor 0.88 for xylose and arabinose and 0.9 for galactose and mannose

### Extraction of xylan and separation from lignin

#### Thermochemical pretreatment with alkali combined with autoclaving

**Fig. 2:**
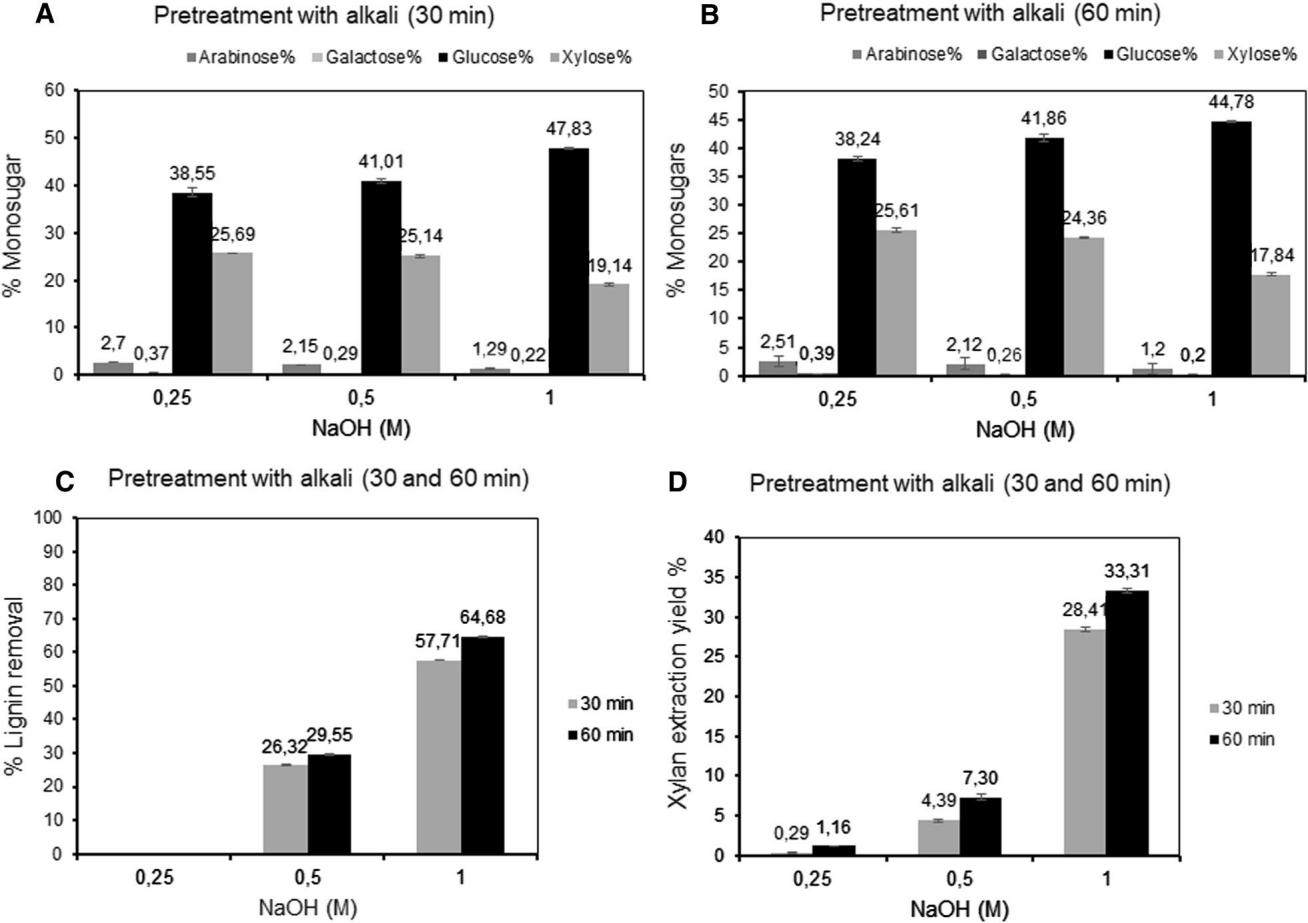
Components from sugarcane bagasse shown as % after extraction using 0.25, 0.5 and 1 M NaOH at 121 °C: **A** composition of monosaccharides in the remaining residue after 30 min extraction; **B** composition of monosaccharides in remaining residue after 60 min extraction; **C** the extracted lignin (% of total lignin); **D** the extracted xylan yields (% of total xylan)

The SCB was pretreated with 0.25, 0.5 and 1 M of NaOH at 121 °C (1 bar) for 30 and 60 min to investigate the effect of the alkali concentration and reaction time on the xylan and lignin solubilization. As seen in Fig. 2A–B, the concentration of alkali exhibited a significant influence on the glucose/xylose ratio in the remaining residue, with higher relative amount of glucose in the insoluble fraction after extraction with higher concentration of alkali. Solubilization of xylan from SCB thus increased with increasing concentration of alkali (Fig. 2D). The 30 min treatment with 0.25, 0.5 and 1 M NaOH released xylan fractions with a dry weight corresponding to 0.08 ± 0.07, 1.12 ± 0.28 and 7.22 ± 0.26% of the dry starting material, respectively, solubilizing 0.29 ± 0.09, 4.39 ± 0.2 and 28.4 ± 0.50% of the xylan present in the SCB (*p*-value < 0.0002). Extension of the treatment to 60 min, resulted in a small increase in solubilization, with xylan fractions corresponding to 0.30 ± 0.09, 1.86 ± 0.19 and 8.45 ± 0.22% of the dry starting material, which is 1.16 ± 0.37, 7.30 ± 0.74 and 33.31 ± 0.88% of the xylan content, respectively (*p*-value < 0.00004). After the treatment, a solid fraction enriched in cellulose was left. The increase in NaOH concentration from 0.25 to 1 M also resulted in co-extraction of lignin, from zero (using 0.25 M NaOH) to 57.7 ± 0.21% of the total lignin after 30 min and to 64.7 ± 0.52% after 60 min pretreatment with 1 M NaOH (Fig. 2C). The sugar composition of the obtained xylan was not significantly affected by the increased extraction time (Table 3).

**Table 3:** Monosaccharide composition (%) and recalculation to polysaccharides in the remaining residue after 30 and 60 min extraction

NaOH (N)	Arabinose	Galactose	Glucose	Xylose	Cellulose	Hemicellulose
Residence time, 30 min						
0.25	2.70 ± 0.01	0.37 ± 0.0	38.55 ± 0.83	25.69 ± 0.12	34.70 ± 0.74	25.32 ± 0.07
0.5	2.15 ± 0.01	0.29 ± 0.05	41.01 ± 0.47	25.14 ± 0.36	36.91 ± 0.42	24.28 ± 0.28
1	1.29 ± 0.03	0.22 ± 0.01	47.83 ± 0.15	19.14 ± 0.25	43.05 ± 0.14	18.18 ± 0.26
Residence time, 60 min						
0.25	2.51 ± 0.07	0.39 ± 0.02	38.24 ± 0.43	25.61 ± 0.41	34.42 ± 0.38	25.10 ± 0.09
0.5	2.12 ± 0.08	0.26 ± 0.01	41.86 ± 0.66	24.36 ± 16	37.67 ± 0.60	23.54 ± 0.19
1	1.20 ± 0.03	0.2 ± 0.0	44.78 ± 0.19	17.84 ± 0.33	40.30 ± 0.17	16.93 ± 0.22

The cellulose and hemicellulose content was calculated based on the monosaccharide composition as explained in Table 2

**Fig. 3:**
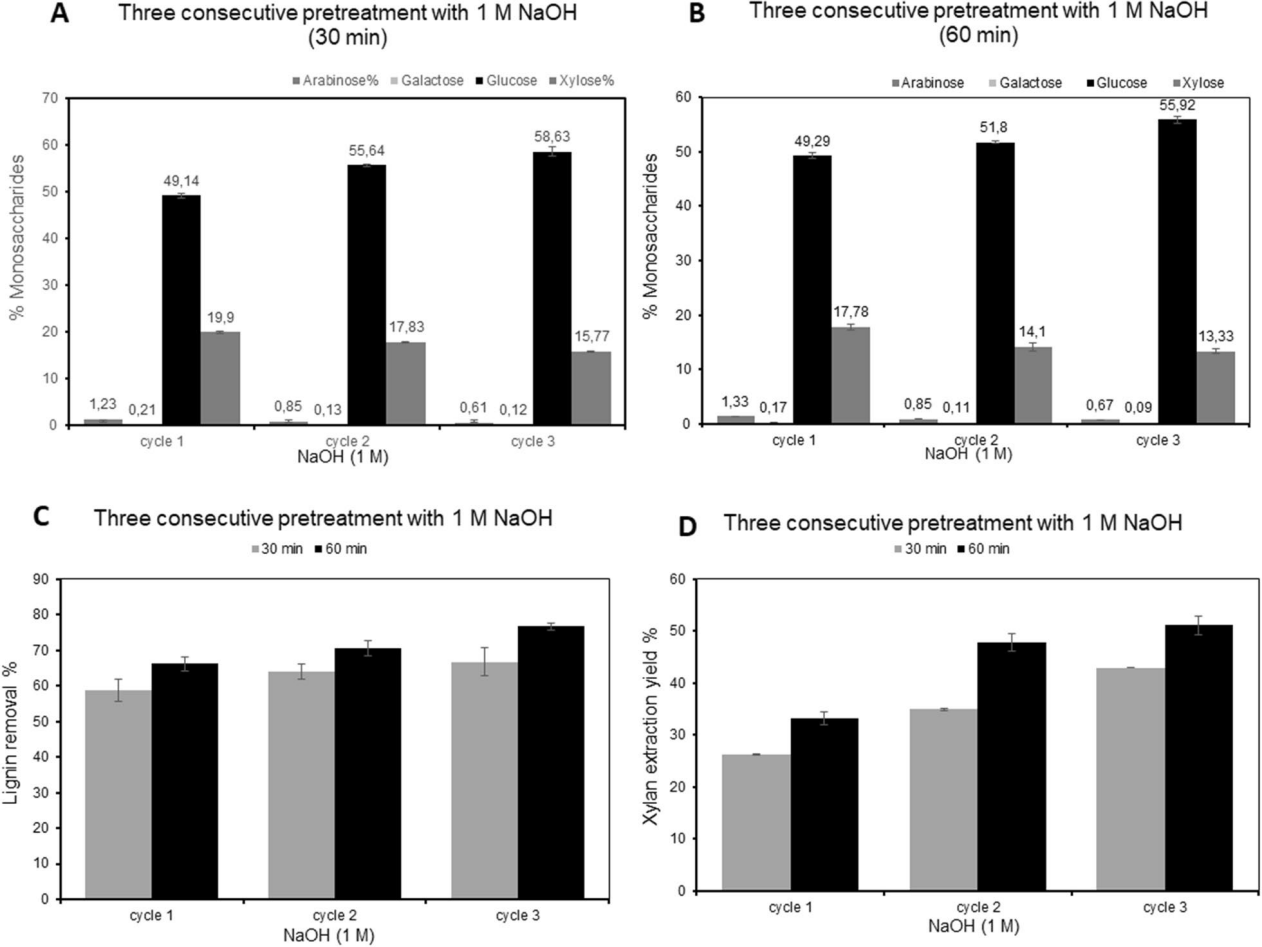
Three consecutive pretreatment with 1 M NaOH at 121 °C: **A** composition of monosaccharides in the remaining residue after 30 min extraction; **B** composition of monosaccharides in the remaining residue after 60 min extraction; **C** the extracted lignin (% of total lignin); **D** the extracted xylan yields (% of total xylan)

Introduction of three consecutive extractions with 1 M NaOH, further increased the fraction of extracted xylan (monitored as dry weight) (Fig. 3A, B and D) which after repeated 30 min treatments, resulted in an accumulated release of 6.7 ± 0.10, 8.8 ± 0.21 and 10.9 ± 0.01% of the dry starting material, after the first, second, and third extraction respectively, corresponding to 26.3 ± 0.41, 35.0 ± 0.84 and 42.9 ± 0.03% of the xylan in the raw material (*p*-value < 0.0002). Three repeats of the 60 min treatment increased the extraction yield to an accumulated weight corresponding to 8.4 ± 0.55, 12.1 ± 0.68 and 13.0 ± 0.45% of the dry starting material (corresponding to 33.2 ± 2.2, 47.8 ± 2.7 and 51.1 ± 1.8% of the xylan in the raw material (*p*-value < 0.008)). The accumulated lignin release in the respective treatment was in this case 66.7 ± 3.9 and 76.7 ± 1.0% of the total lignin present in the raw material after 30 and 60 min repeated pretreatments, respectively (Fig. 3C), showing that the ratio between xylan and lignin remained the same, irrespective of the extension in time to 60 min. The xylose/arabinose ratio is increasing in the remaining solid residue after each extraction, showing that the substitution degree is reduced in the residuals after each extraction (Table 4).

**Table 4:** Sequential extraction

NaOH 1 M	Arabinose	Galactose	Glucose	Xylose	Cellulose	Hemicellulose
Reaction time, 30 min						
Cycle 1	1.23 ± 0.07	0.21 ± 0.02	49.14 ± 0.45	19.90 ± 0.20	44.23 ± 0.4	18.72 ± 0.10
Cycle 2	0.85 ± 0.01	0.13 ± 0.0	55.64 ± 0.20	17.83 ± 0.16	50.08 ± 0.18	16.51 ± 0.21
Cycle 3	0.61 ± 0.06	0.12 ± 0.03	58.63 ± 0.99	15.77 ± 0.01	52.77 ± 0.89	14.49 ± 0.01
Reaction time, 60 min						
Cycle 1	1.33 ± 0.06	0.17 ± 0.04	49.29 ± 0.51	17.78 ± 0.52	44.36 ± 0.46	16.97 ± 0.55
Cycle 2	0.85 ± 0.03	0.11 ± 0.0	51.80 ± 0.25	14.10 ± 0.75	46.62 ± 0.22	13.25 ± 0.68
Cycle 3	0.67 ± 0.04	0.09 ± 0.0	55.92 ± 0.69	13.33 ± 0.48	50.33 ± 0.62	12.40 ± 0.45

Monosaccharide composition (%) and recalculation to polysaccharides in the remaining residue. Data after each extraction (30 and 60 min, respectively) in totally three repeats using 1 M NaOH at 121 °C are shown. The cellulose and hemicellulose content was calculated from the monosaccharide composition as described in Table 2

**Fig. 4:**
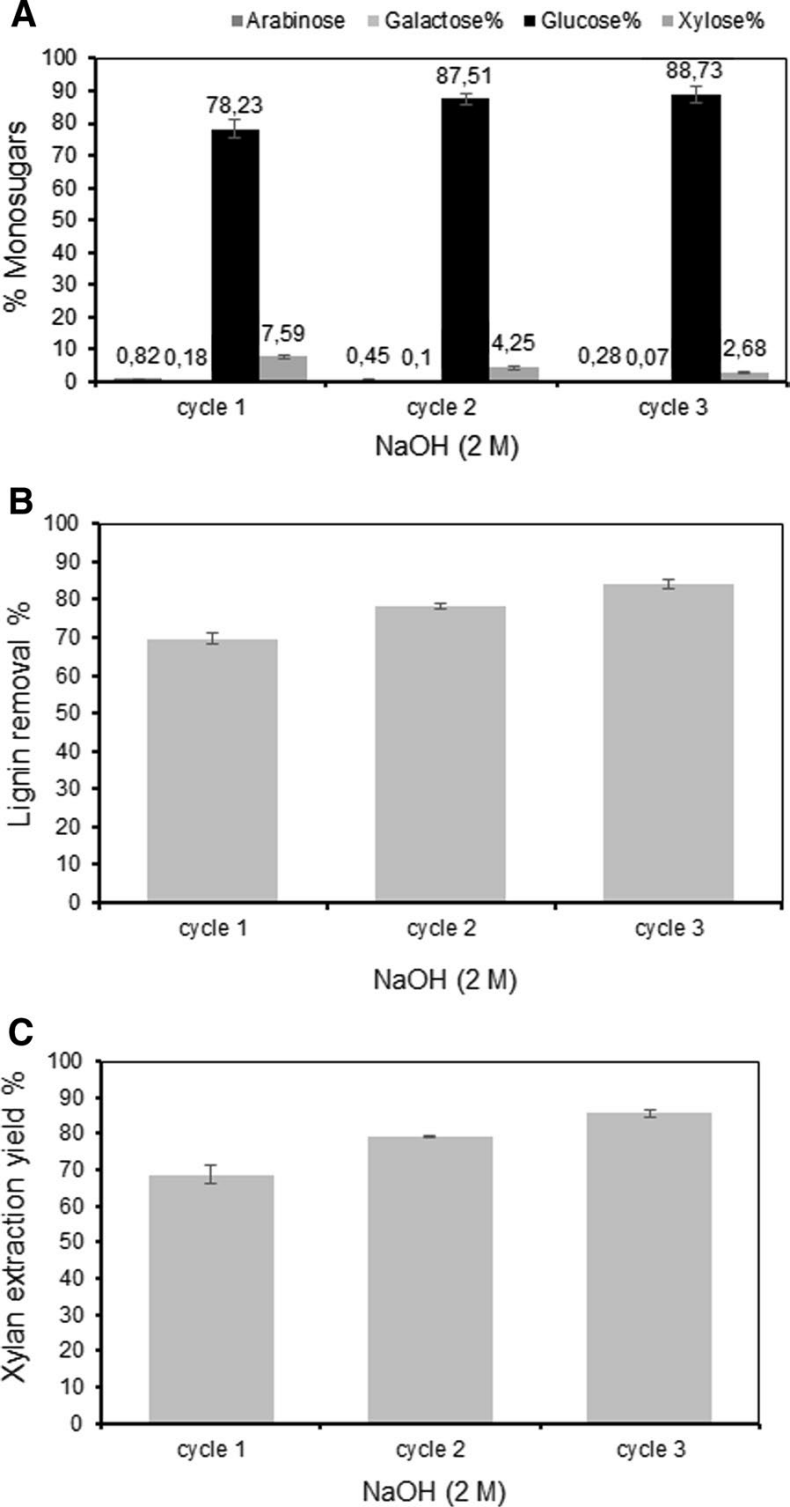
Three consecutive extractions with 2 M NaOH at 121 °C for 60 min: **A** composition of monosaccharides in the remaining residue; **B** the extracted lignin (% of total lignin); **C** the extracted xylan yields (% of total xylan)

Both NaOH concentration and time were thus shown to influence the xylan extraction efficiency at the selected temperature (121 °C). The increase in xylan yields with time showed that a longer extraction time increased the overall yields, but that this also led to a corresponding co-extraction of lignin. The extraction time was, however, not as significant as the concentration of alkali for releasing xylan. To investigate the effect of a further increase in NaOH concentration the consecutive experiment was repeated using 2 M NaOH, and 60 min extraction time (Fig. 4). This condition led to release of 17.4 ± 0.62, 20.1 ± 0.04, and 21.7 ± 0.28% of the dry starting material, which based on the xylan content in the material would correspond to an accumulated yield of 68.7 ± 2.46, 79.3 ± 0.17, and 85.6 ± 1.10% of the xylan in the bagasse (*p*-value < 0.004). This repeated pretreatment also solubilized 69.65 ± 0.21, 78.31 ± 0.35, and 84.13 ± 0.33% of the lignin.

To separate xylan and lignin, xylan was subsequently precipitated with ethanol and washed, while most of the lignin remained in the liquid phase (Fig. 1). A significant amount of the lignin is thus released at NaOH concentrations of 1 M and higher, and is kept in the liquid phase after the downstream precipitation step, allowing separate use of xylan and lignin. Calculation of the lignin content showed that 80% of the solubilized lignin was kept in the liquid phase after precipitation, also motivating capture of this stream in a biorefinery perspective. Although outside the scope of this study, capture of lignin from this step could be explored by membrane technologies [52], to allow further valorization of this material.

An added advantage of the 2 M NaOH extraction procedure was that this resulted in a relatively pure residual cellulose fraction (see further below). The cellulose purity in all samples of sugarcane bagasse pretreated with 2 M NaOH was twice as high as in the raw material due to removal of large amounts of both lignin and hemicellulose.

Xylan yields in the same range as in this work was reported by Jayapal et al. [40], who obtained 85% xylan recovery, although using even higher concentration of NaOH (12% or 3 M) combined with steam processing. Peng et al. [45], have reported relatively high yields using a different approach, applying sequential extractions of hemicellulose from bagasse with increasing concentration of NaOH in the sequence, starting with water, then using 1% (0.25 M) NaOH and finally 3% (0.75 M) NaOH at 50 °C for 3 h in each round of extraction. This approach released 14.3, 32.5, and 28.1% of the hemicellulose in each round of extraction (releasing 74.9% of the hemicellulose), and 2.2, 31.5, and 20.4% of the lignin (releasing 54.1% of the lignin) [45], which is slightly lower than achieved here, but using a lower amount of NaOH in the process.

#### Pressurized liquid extraction (PLE)

To explore the possibility to decrease the concentration of NaOH by introducing high pressure (100 bar), pressurized liquid extraction (PLE) was chosen as an extraction technology, previously reported as a green alternative [53]. Another interest was to investigate if the high pressure would change the ratio between extracted xylan and lignin.

An experimental design (Box–Behnken type) was set up, varying the three parameters temperature (50, 100, and 150 °C), alkali concentration (0, 0.05 and 0.1 M NaOH) and extraction time (10, 20 and 30 min) at three levels. The model fitting plots (Additional file 1: Figure S1) show that the values of R^2^ are greater than 0.9 and Q^2^ ranges between 0.6 and 0.8 which indicates valid models and the quadratic equation well represents the system under the given experimental domain. The coefficient plots indicate that alkali concentration and temperature have direct and positive influence on solubilizing xylan while decreasing solubilization of cellulose. The contour plots (Additional file 1: Figure S2, showing remaining residues in the solids) revealed that, at the conditions chosen, high temperature and high NaOH concentration enhanced solubilization of xylan (i.e., resulted in lower residual amounts) and decreased solubilization of cellulose (i.e., higher concentration in the residuals). Long extraction time at high temperature and high NaOH concentration significantly increased lignin solubilization. An optimizer function, based on a simplex algorithm with a non-linear desirability, was applied to find conditions with minimum responses of hemicellulose and lignin, combined with a maximum response of cellulose. The best condition was found to be at 150 °C, 0.1 M NaOH, with an extraction time of 22 min.

The extraction yields of xylan at the proposed conditions were however low, with a maximum yield of 16.7% [experiment No.4 (150 °C, 0.1 M NaOH, 20 min), Table 5]. However, this yield is higher than what was obtained using 0.5 M NaOH in a single 60 min extraction at 1 bar (which yielded 7.3 ± 0.74% of the xylan, see also above), showing that the increased pressure indeed reduced the need of alkali. A higher severity factor (increased temperature or alkali concentration which was not possible in our study as that might damage the PLE equipment) is, however, probably necessary to release more xylan from SCB. A further increase in the extraction temperature (to 180–190 °C) reported by Kilpeläinen et al [54] showed to be useful to reach higher yields in extractions of xylan from birch sawdust. Lignin was, however, efficiently extracted at the conditions used here (experiment 4, Table 5), showing that PLE may be considered for early lignin removal, as only 13% lignin was remaining in the residual residue after the extraction.

**Table 5:** Pressurized liquid extraction (PLE) experimental design

PLE	NaOH (M)	Temp	Time	Ara	Gal	Glu	Xyl	Lignin	Cellulose	Hemicellulose	Xylan extraction yield (% of total xylan)
1	0.05	150	10	1.28	0.26	53.60	23.61	19.31	48.24	22.14	12.82
2	0.05	50	10	1.64	0.54	40.96	22.77	25.93	36.86	21.97	13.49
3	0	150	20	0.73	0.34	45.62	24.33	27.84	41.06	22.36	11.94
4	0.1	150	20	0.92	0.18	60.46	22.92	13.05	54.41	21.14	16.74
5	0	100	10	1.52	0.54	42.28	24.13	28.35	38.06	23.05	9.21
6	0	50	20	1.43	0.51	41.52	23.53	25.82	37.36	22.42	11.69
7	0.1	50	20	1.92	0.51	42.38	23.01	25.18	38.14	22.40	11.78
8	0.1	100	30	1.63	0.39	49.88	24.35	18.44	44.89	23.21	8.59
9	0.05	100	20	1.84	0.46	45.83	25.36	23.68	41.25	24.35	4.08
10	0	100	30	1.46	0.52	42.25	24.19	23.98	38.03	23.04	9.24
11	0.05	50	30	1.59	0.52	42.12	23.30	29.50	37.91	22.38	11.87
12	0.05	100	20	1.78	0.44	45.59	24.52	23.47	41.03	23.97	5.61
13	0.1	100	10	1.77	0.44	48.31	24.45	20.20	43.48	23.47	7.58
14	0.05	150	30	1.15	0.21	53.58	23.68	17.08	48.22	22.04	13.21
15	0.05	100	20	1.89	0.48	45.21	25.44	23.35	40.69	24.48	3.58

Conditions, chemical composition of monosaccharides (%) and polymeric sugar contents in the remaining residue, and extracted xylan yields (with three replicates in experiments are shown. No.9, 12 and 15 with STDV 1.05 in xylan extraction yield). The cellulose and hemicellulose were calculated from the monosaccharide yields as explained in Table 2

*Ara* arabinose, *Gal* galactose, *Glu* glucose, *Xyl* xylose

#### Monosaccharide composition in extracted xylan fractions

To investigate the influence of the extraction conditions on the obtained xylan structure, eight xylan fractions from the various trials were selected for composition analysis. These included the three 60 min cycles (121 °C, 1 bar) extracted by 2 M NaOH, and by 1 M NaOH, respectively, as well as the 60 min extraction by 0.5 M NaOH (121 °C, 1 bar), and the 20 min PLE extraction by 0.1 M NaOH, at 100 bar, 150 °C. The xylan obtained after ethanol precipitation in the respective fraction was hydrolyzed and the monosaccharide composition was determined (Table 6).

**Table 6:** Chemical composition (%) of purified extracted xylans

Picture of xylan fraction and sample abbreviation		Ara	Gal	Glu	Xyl	Man	UA	Xyl/Ara	Xyl/UA
	2 M-C1	6.72 ± 0.08	0.27 ± 0.01	7.8 ± 0.44	75.0 ± 1.21	0.43 ± 0.05	3.3	11.18 ± 0.31	22.74 ± 0.22
	2 M-C2	5.64 ± 0.10	0.31 ± 0.05	5.97 ± 0.51	67.4 ± 1.27	nd	2.46	11.94 ± 0.02	27.38 ± 1.46
	2 M-C3	4.64 ± 0.09	0.11 ± 0.04	3.97 ± 0.12	65.1 ± 0.54	nd	1.3	14.05 ± 0.16	50.1 ± 2.05
	1 M-C1	7.93 ± 0.13	0.41 ± 0.03	12.27 ± 0.33	73.0 ± 1.0	0.56 ± 0.03	2.48	9.20 ± 0.02	29.42 ± 0.36
	1 M-C2	6.83 ± 0.21	0.29 ± 0.0	6.7 ± 0.21	76.4 ± 0.81	nd	2.68	11.19 ± 0.22	28.5 ± 0.60
	1 M-C3	5.32 ± 0.11	0.21 ± 0.01	4.56 ± 0.19	72.2 ± 1.12	nd	4.05	13.57 ± 0.08	17.82 ± 0.04
	0.5 M-60 min	7.48 ± 0.12	0.6 ± 0.03	21.88 ± 0.67	48.5 ± 1.12	0.28 ± 0.01	2.76	6.49 ± 0.05	17.58 ± 0.04
	PLE (No.4)	6.63 ± 0.08	0.49 ± 0.04	13.14 ± 0.15	58.1 ± 0.76	0.51 ± 0.04	3.61	8.76 ± 0.0	16.09 ± 0.23
	Untreated bagasse	2.24 ± 0.07	0.72 ± 0.0	36.58 ± 0.13 ara>	25.7 ± 0.79	0.16 ± 0.05	1.87	11.18 ± 0.31	13.75 ± 0.51

2 M-C1: 1st cycle extraction in 2 M NaOH; 2 M-C2: 2nd cycle extraction in 2 M NaOH; 2 M-C3: 3rd cycle extraction in 2 M NaOH; 1 M-C1: 1st cycle extraction in 1 M NaOH, 1 M-C2: 2nd cycle extraction in 1 M NaOH; 1 M-C3: 3rd cycle extraction in 1 M NaOH, 0.5 M-60 min: 60 min extraction in 0.5 M NaOH; PLE(No4): pressurized liquid extraction using 0.1 M NaOH, at 150 °C, 20 min

*Ara* arabinose, *Gal* galactose, *Glu* glucose, *Man* mannose, *Xyl* xylose, *UA* uronic acids, *nd* not detected

**Fig. 5:**
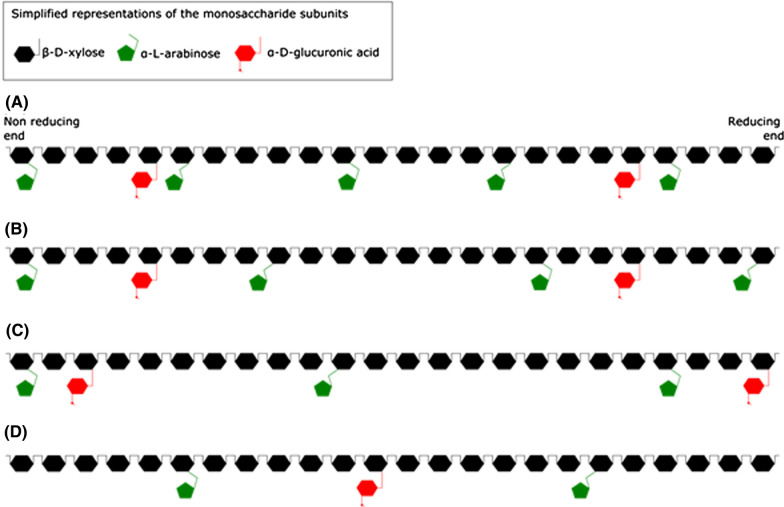
Hypothetical structures of xylan fragments extracted from sugar cane. It is based on the monosaccharide analysis (see Table 6) and assuming glycoside bonds common in different types of xylan. The backbone is linked by β-(1–4) glycosidic bonds; arabinoses can be linked to the xylan backbone by α-(1–2) or α-(1–3) bonds; while, glucuronic acids are linked by α-(1–2) bonds. Notice that the branching pattern is affected by the extraction conditions. **A** Extraction with 0.5 M NaOH for 60 min. **B** Pressurized liquid extraction (experiment No.4). **C** Extraction with 2 M NaOH in a single or two cycles. **D** Extraction with 2 M NaOH and in three cycles

The major monosaccharide in all extracted xylans was xylose, which dependent on the extraction conditions comprised 48.5 ± 1.12–76.4 ± 0.81% of the total monosaccharides in the different fractions (Table 6). Varying amount of glucose (4.0 ± 0.12–21.9 ± 0.67%) was observed, indicating some cellulose solubilization. In addition, smaller amounts of arabinose (5.3 ± 0.11–7.9 ± 0.13%), uronic acids (1.3 ± 0.04–4.05 ± 0.8%) and galactose (0.11 ± 0.04–0.6 ± 0.03%) were verified in the eight extracted xylan fractions, confirming presence of substituents. Some variation in the xylose/arabinose and xylose/uronic acid ratio is observed (Table 6), leading to slightly different predicted xylan structures (Fig. 5). The xylan should, however, according to the scheme for naming xylan developed by Ebingerova [55], based on the ratio of arabinose and glucuronic acid, in all cases be classified as glucuronoarabinoxylan (GAX). Variation in substituent groups of SCB xylan has previously been shown to depend on both the SCB variety [56] and the conditions of the extraction method [57]. In our study, it is clear that the conditions affected the substituent content. The xylose/arabinose ratio in extracted xylans increased from 11.2 ± 0.31 to 14.1 ± 0.16 (in the 2 M NaOH consecutive process) and from 9.2 ± 0.02 to 13.6 ± 0.08 (in the 1 M NaOH consecutive process) with increasing treatment cycles. This suggested that the degree of arabinose branching decreased in every cycle. Also, the degree of arabinose branching decreased with increasing alkali strength, which in the analyzed samples show highest arabinosylation in the 60 min, 0.5 M NaOH extraction. No such clear trend was seen for the glucuronic acids, but their content clearly varied in the different fractions.

Xylans in SCB have previously been defined as acetylated glucurono-arabinoxylans (GAX) with a β-1,4 linked xylose backbone carrying substitutions at the positions C2 and C3 including arabinosyl, uronic acid and acetyl groups [35, 46, 58, 59]. The xylose content of the polymer has a reported range from 43 to 93%, with arabinose being the most common substituent linked to the xylan backbone by α-1,2 or α-1,3 linkages [12]. This is in accordance with the xylose range and arabinose content in the different fractions of this work (Table 4, Fig. 5). Galactose was also found in minor amounts (Table 4), and has been reported to be linked by a β-1,5 linkage to the xylan backbone [12]. Acetyl groups were, however, not found in any of the extracted xylan fractions in this work.

#### Molecular weight determination and FT-IR spectra of selected xylan fractions

The molecular weights of the xylan extracted and purified from three fractions (1^st^ extraction by 2 M NaOH [2 M-C1], 1^st^ extraction by 1 M NaOH[1 M-C1] and by 0.5 M NaOH [0.5 M-60 min], in all cases using 60 min procedures) were determined by size exclusion chromatography. The results showed one major broad peak for the two fractions, 2 M-C1 and 1 M-C1, with a retention time of 276.5 min corresponding to a molecular weight of 212 kDa (Additional file 1: Figure S3). This molecular weight is higher than previously reported from other studies of sugarcane bagasse xylan: (65–86 kDa) [12] or (7–45 kDa) [35], which either indicate xylan of a higher molecular weight, or xylan interacting (or aggregating) with other chains or with lignin. The range of molecular weights observed for xylans however varies broadly. For example, arabinoxylan from wheat bran is reported in a molecular weight range of 470–600 kDa [60] while xylan extracted from corn stover have a reported molecular weight of 49 kDa [61]. The fraction from the 0.5 M NaOH extraction, also showed a broad peak, but with a maximum at a lower molecular weight, 47 kDa (Additional file 1: Figure S3). This suggests that the lower concentrations of alkali solubilized more of smaller molecular sized xylans and that increased alkali concentration led to interactions, and apparent higher molecular weight xylans.

**Fig. 6:**
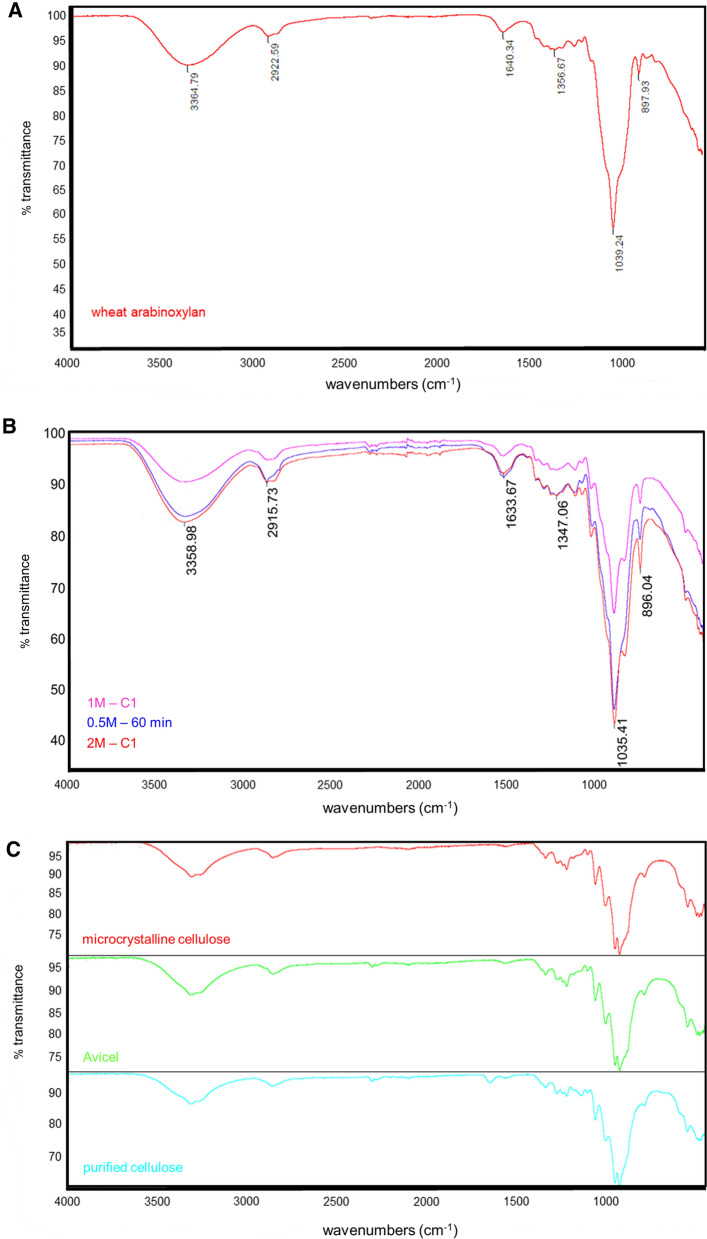
**A** FT-IR spectrum of commercial wheat arabinoxylan (Megazyme). **B** FT-IR spectra of the three fractions of extracted xylans, 2 M-C1 (first extraction by 2 M NaOH during 60 min), 1 M-C1 (first extraction by 1 M NaOH during 60 min) and 0.5 M-60 min (extraction by 0.5 M NaOH during 60 min). All extractions at 121 °C. **C** FT-IR spectra of purified cellulose and the two commercial celluloses Avicel, and microcrystalline cellulose

FT-IR spectra of the same three xylan fractions, (2 M-C1, 1 M-C1 and 0.5 M-60 min) showed that the three fractions clearly illustrated the typical signal pattern for xylans (Fig. 6). A major peak at 1035 cm^−1^ was corresponding to vibrations of the C–O, C–C and C–OH stretching [57, 62]. The peak at 896 cm^−1^ indicated glycosidic linkages in the xylan backbone [63]. The absorption at 1633 cm^−1^ is attributed principally to the water absorbed by xylans. The C–H stretching vibration gave a signal at 2915 cm^−1^. The band at 3358 cm^−1^ corresponded to the hydroxyl stretching vibrations of xylans, as well as the water involved in the hydrogen bonding [59].

### Cellulose purification and FT-IR spectra

Alkali extraction is the most efficient method for separating cellulose from the various biomass sources by releasing a large amount of hemicellulose polysaccharides in alkaline solution [29]. In this study, after three consecutive pretreatments of bagasse with 2 M NaOH at 121 °C for 60 min, the remaining solid residue had a high content of cellulose, consisting of 80% glucose. Therefore, this fraction was acid purified (Fig. 1, see also methods), resulting in cellulose of high purity, with a sugar composition of 95.8 ± 0.59% glucose, with only minor amounts of xylose (3.1 ± 0.13%) (Additional file 1: Figure S4). The remaining amount of lignin in the cellulose was determined to 1.8 ± 0.07%. This material, can either be used in polymeric form, or be hydrolyzed to glucose residues, e.g., for application as carbon source in ethanol production.

The FT-IR spectrum of the extracted cellulose is very similar to the spectra of microcrystalline cellulose and Avicel (Fig. 6). A minor difference can be seen in intensity around the peak at 1729 cm^−1^, which is judged to correspond to vibration of water molecules absorbed in cellulose. The broad peak at 3333 cm^−1^ is characteristic for stretching vibration of the hydroxyl group in cellulose. The band at 2898 cm^−1^ is attributed to CH stretching vibration of all hydrocarbon constituent in polysaccharides and the band at 1054 cm^−1^ is assigned to the C–O stretching vibration. The band at 1428 cm^−1^ is associated with the amount of the crystalline structure of the cellulose, while the band at 897 cm^−1^ is assigned to the amorphous region in cellulose [64–66]. Peaks around 1500 cm^−1^ are defined for aromatic backbone in lignin [67] and clearly no such peaks are present in the purified cellulose fraction.

### Enzymatic production of xylo-oligosaccharides

Xylans have lately gained interest for the development of protocols for XOS production, for applications as prebiotic food ingredients, and XOS are regarded as safe for use by both FDA and EFSA [21]. XOS with a degree of polymerization (DP) of 2–5 are often preferred in functional food production owing to the utilization of these compounds by probiotic microorganisms [68], and within this range XOS with an average DP of 2–3 (especially xylobiose (X2)) gain importance as they present faster fermentation kinetics in the probiotic microorganisms that can promote a favorable intestinal environment [69].

**Fig. 7:**
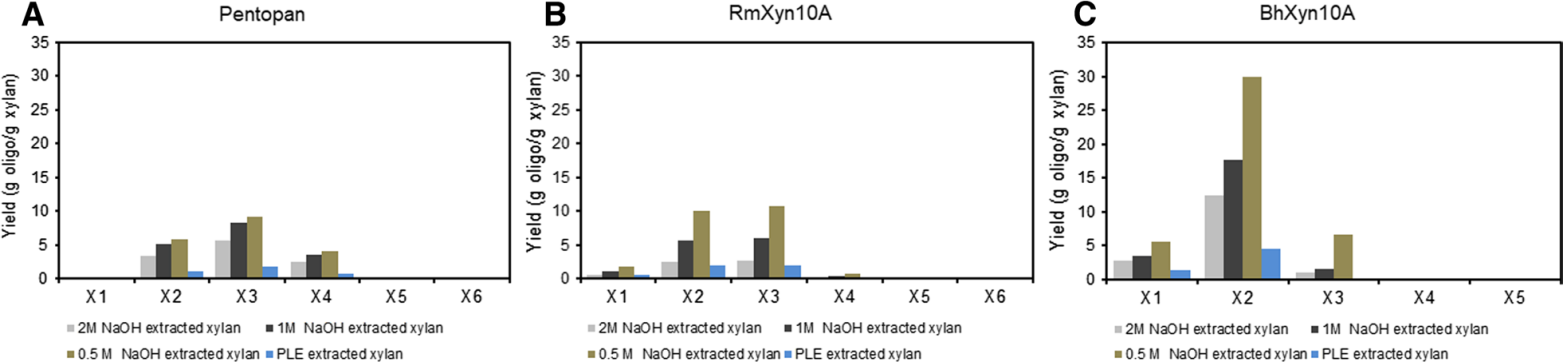
Enzymatic hydrolysis of the extracted xylans: **A** Pentopan; **B** RmXyn10A; **C** BhXyn10A

**Fig. 8:**
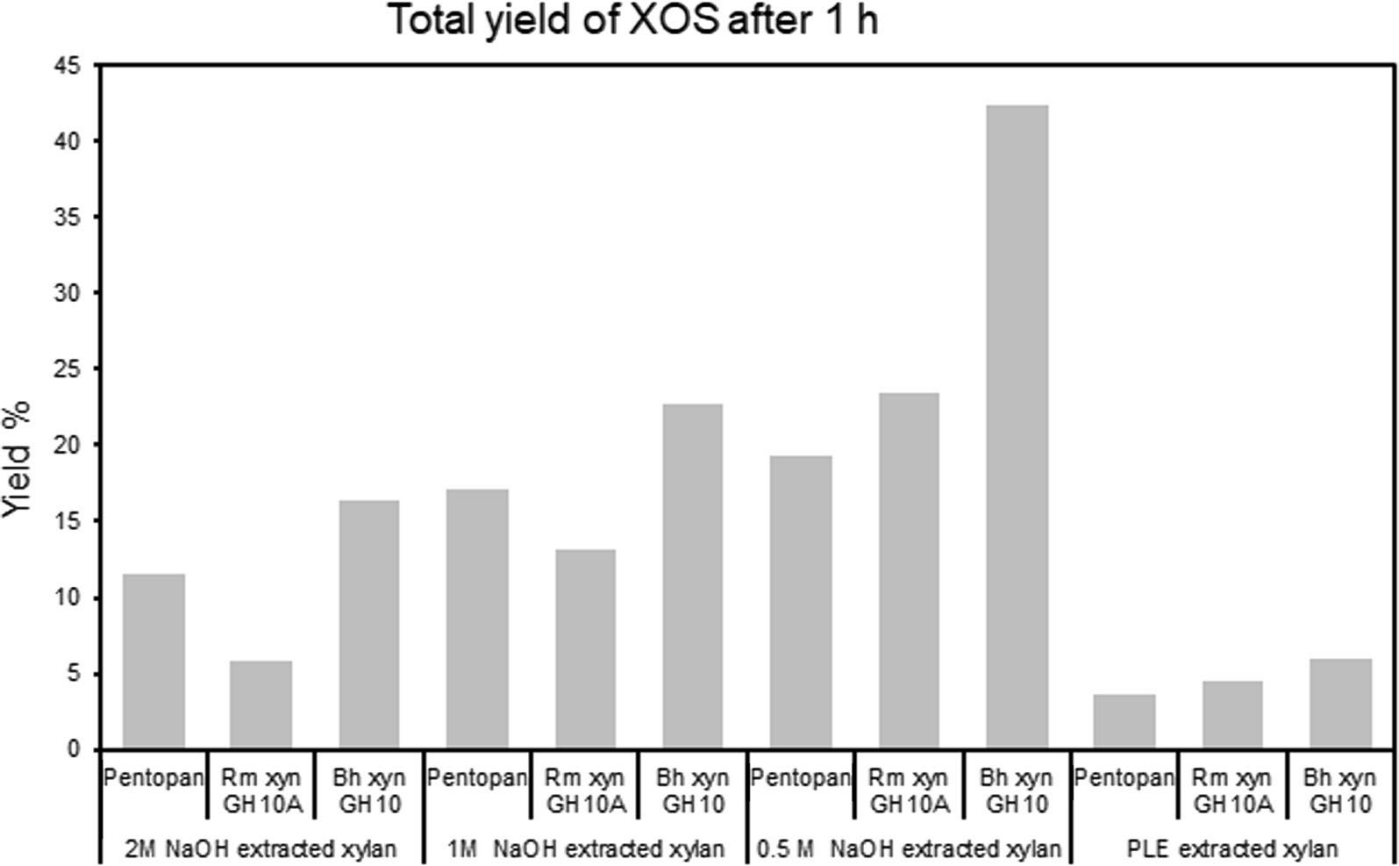
Total yield of produced xylo-oligosaccharides (XOS) after 1-h incubations at 62 °C

Thus far, only limited attention has been given to SCB xylan in the field of XOS production, despite its large availability. Zhang et al. [70], has used a thermo-chemical/physico-chemical study in which birch and SCB is mixed to utilize autohydrolysis where acetyl groups released from the birch xylan aids in converting xylan to oligosaccharides. Dai et al., used a treatment with furoic acid to obtain XOS from SCB [71]. Enzymatic hydrolysis, is another mild processing alternative to gain XOS. For SCB, this has previously been explored using native crude mixes of xylanases from *P stipidis*, on alkaline extracted xylan (using KOH 10%) from SCB [49] or enzyme mixes from *Thermoascus aurantiacus* [50]. The present study explored the use of three individual enzymes from glycoside hydrolase family 10 (GH10) and 11 (GH11) for XOS production in a screening procedure comparing the xylan fractions obtained using the different alkaline conditions. For this purpose the endoxylanases *Rm*Xyn10A, and *Bh*Xyn10A (from GH10) and the commercially available GH11 enzyme Pentopan (from Novozymes), were screened using four fractions of purified extracted xylans (2 M-C1 (with a Xyl/Ara ratio of 11, and Xyl/UA ratio of 23), 1 M-C1 (with a Xyl/Ara ratioof 9, and Xyl/UA ratio of 29), 0.5 M-60 min (a Xyl/Ara ratio of 6, and Xyl/UA ratio of 17) and PLE No.4 (with a Xyl/Ara ratio of 9, and Xyl/UA ratio of 16) (Table 6). Maximum enzymatic conversion yields, for all enzymes in the screen, were obtained using the low molecular weight xylan, extracted by 0.5 M NaOH during 60 min, which after 1 h incubation gave a maximum yield of 42.29 ± 1.7% XOS, using *Bh*Xyn10A (Figs. 7 and 8). The predominant XOS produced was xylobiose, followed by xylotriose (Fig. 7). The lowest total production of XOS was found in PLE extracted xylan and in the xylan extracted by 2 M NaOH (Fig. 8). These two xylan fractions represent one of the more substituted (PLE(No4), Table 6), and one less substituted xylan (2 M-C1, Table 6), showing that the type of branching is not the major bottleneck here. Both these fraction however, displayed the highest amount of co-extracted lignin, and xylan of apparent high molecular weight, which indicates that interactions with lignin occur that may pose a problem for efficient enzymatic conversion. This is in line with previous conclusions by Lin and coworkers [72] who proposed that adsorption of lignin on the enzyme, reduced the hydrolysis, but that this could be circumvented by introduction of surfactants in the system.

## Conclusion

Integration of processing techniques to utilize different parts of the material is of increasing importance for efficient biomass utilization of industrial residues and byproducts. In this work, xylan is extracted from SCB, with separation of lignin and cellulose, allowing retrieval of the different materials in separate fractions. The present results clearly show that repeated extraction with high concentration of alkali (in this case 2 M NaOH), is an efficient way to release xylan from sugarcane bagasse (85.6 ± 1.10% of the xylan was released), which by precipitation separates it from the major part of the lignin stream, and that this gives the possibility to also obtain a clean cellulose fraction of high purity (95.8 ± 0.59% of cellulose) after acid purification of the remaining residue. Use of high concentration of alkali for the xylan extraction reduces the substitution degree on the xylan, especially the arabinosylation. Lignin co-extracts with xylan at high concentration of alkali, but if high pressure was applied (using PLE), lignin appeared easier to release than xylan, at lower alkali concentration (0.1 M NaOH). Thus, dependent on the extraction conditions, there could be a possibility to separate lignin either before or after xylan solubilization. Our study showed that enzymatic hydrolysis was more efficient using a fraction of xylan extracted at lower alkaline strength (0.5 M NaOH for 60 min at 121℃, where a maximum conversion yield to XOS was 42.3 ± 1.7%). For this purpose, it may hence be good to compromise the overall yield to obtain a fraction for enzymatic hydrolysis, while higher severity may be used to obtain polymeric xylan for other purposes. This clearly shows the benefit of sequential extractions to maximize the yield, but with the possibility to utilize variants from different stages in the process in different applications.

## Methods

### Materials

Sugarcane bagasse (SCB) was provided from a sugar factory in Iran, Khuzestan Province (haft tapeh). It was first dried at room temperature and was then ground using a laboratory mill to pass a 0.5-mm sieve and stored under darkness at room temperature. All chemicals were supplied from Sigma-Aldrich unless otherwise specified. Guluronic acid was provided from Chemos (Germany). Sulfuric acid (72%) was purchased from Thermo Fisher (Germany). Sodium hydroxide (NaOH) pellets and acetic acid (glacial) 100% were supplied by Merck (Darmstadt, Germany). Ethanol (99%) was purchased from Solveco Group (Rosenberg, Sweden). Ultrapure water (18,2 MΩ/cm) was provided through a Milli-Q instrument (Millipore, Billerica, MA, USA). Oligosaccharide standards (xylose, xylobiose, xylotriose, xylotetraose, xylopentaose and xylohexaose) were provided from Megazyme (Ireland). Standards for FT-IR analysis, include Wheat Arabinoxylan (Megazyme), Avicel (Sigma-Aldrich) and microcrystalline cellulose; from Cellupharm (Sweden) AB.

### Extraction of xylan

#### Single thermo-chemical pretreatment by alkali and autoclaving

The pretreatment was carried out using various concentrations of NaOH. The solid to liquid ratio was 1:10 (w/v). Five grams of sugarcane bagasse were suspended in 50 mL of 0.25 M, 0.5 M, and 1 M NaOH, separately and autoclaved (Inspecta, Thermo Scientific) at 121 °C (1 bar) for 30 or 60 min. After autoclaving, the samples were immediately cooled down to room temperature and vacuum filtered using crucibles with filters to separate solid and liquid fractions. The remaining solid residues were washed with distilled water until samples were neutralized. The solid residues were dried in an oven at 65 °C for further analysis.

#### Three consecutive pretreatments by alkali and autoclaving

Five grams of SCB was treated with 50 mL 1 M NaOH and autoclaved at 121 °C for 30 or 60 min. After autoclaving and cooling down the samples, solid and liquid fractions were separated by vacuum filtration. The extraction was then repeated twice and the remaining solid residues were washed and dried for further analyses. A parallel experiment was carried out using 2 M NaOH at 121 °C for 60 min in three cycles and the remaining solid residues were washed and dried for further analyses.

#### Pressurized liquid extraction (PLE) in the presence of alkali

The PLE was carried out using a Dionex ASE-350 (Thermo Fisher, Germering, Germany). The extractions were started by weighing and loading 0.3 g of ground SCB into a 10 mL stainless-steel extraction vessel. The extraction vessel was fitted with metallic filters (0.45 µm pore size) at the inlet and the outlet. The extractions were conducted in a static mode and after each extraction the vessel was flushed with fresh solvent, in a volume corresponding to 60% of the vessel volume. The remaining material in the vessel was flushed with nitrogen for 90 s.

To find the best extraction conditions, a Box Behnken design with three center points was created in MODDE 10.1 (Sartorius, Stedim Biotech, Malmö, Sweden) and used to examine the effect of extraction temperature (T; 50–150 °C), extraction time (t; 10–30 min) and NaOH concentration (NaOH; 0–0.1 M), on the yield of cellulose, hemicellulose and lignin from ground sugarcane bagasse. Multiple linear regression (MLR) was used to calculate the fitting model and response surface. The adequacy of the models was evaluated by the R^2^ and Q^2^ values (where *R*^2^ shows the model fit and *Q*^2^ shows an estimate of the future prediction precision), predicted vs. observed plot and coefficient plots. The optimum processing conditions for the maximum yield were obtained by using graphical and numerical analysis based on the criteria of the desirability function and the response surface plots. The obtained residues were treated and purified as described in Fig. 1.

### Purification of xylan extracts

After the respective pretreatment, the pH of liquid fraction was adjusted to 5 by glacial acetic acid (99% v/v). Then, xylan was precipitated with an equal volume of ethanol (99% v/v) at 4 ºC overnight. A high ethanol concentration was used to promote obtaining also the branched xylans [73]. Precipitated xylan was separated by centrifugation (Sigma 3-16Pk) for 20 min at 4500 rpm and then washed with ethanol (50% v/v) 3 times. Most of the lignin remained in the liquid phase and was separated from the xylan at the precipitation step. The precipitated xylan was dialyzed in 3 cycles against 5 L milli-Q water using a 10 kDa of cut-off dialysis membrane for 8 h (every cycle) in a cold room. Finally, the samples were frozen at − 80 ºC and freeze dried using a lyophilizer (Labconco Lyph. Lock 12).

### Cellulose purification

The residuals after the third cycle in the three cycles extraction of xylan with 2 M NaOH at 121 °C for 60 min, was used for purification of cellulose. The solids were first dried in an oven (65 °C) then, 40 mL of acetic acid (80%; v/v) and 4 mL of concentrated nitric acid (69%; v/v) were added to 1 g of sample at 120 °C during 20 min. The mix was cooled down and 50 mL of ethanol 99% was added and vortexed. The solid fraction was separated by centrifugation at 4000 rpm for 10 min. The supernatant was decanted, and the solid material was washed with 100 mL of ethanol 99% and centrifuged at 4000 rpm for 10 min. The supernatant was discarded, and the solid fraction was washed with 100 mL Milli-Q and separated by centrifugation at 4000 rpm for 10 min. These washing steps with ethanol and water were repeated one more time. Finally, the excess of water was discarded, and the purified cellulose was frozen at − 80 ºC and freeze dried.

### Enzymatic production of xylo-oligosaccharides

Three endo-xylanases: PENTOPAN (from glycoside hydrolase family 11, GH11, manufactured by Novozymes, resuspended in 100 mM citrate phosphate buffer, 1 mg/mL at pH 5), *Rm*Xyn10ACM (the catalytic domain from a thermostable GH10 xylanase from *Rhodothermus marinus*, supplied in 100 mM sodium phosphate buffer, 3.5 mg/mL at pH 7) and *Bh*Xyn10AH (an thermostable alkali-tolerant GH10 xylanase from *Bacillus halodurans*, supplied in 100 mM glycine NaOH buffer, 5.7 mg/mL at pH 9); were used for enzymatic hydrolysis of extracted xylans for production of xylo-oligosaccharides as described by Salas-Veizaga [62].

Enzymatic hydrolysis of extracted xylans was conducted by adding 200 µL of the xylan substrate solution (0.5% (w/v) dissolved in the same buffer as used for the enzyme) to 2µL of each enzyme (with initial concentration of 0.007 mg/mL) and incubation for 1 h at 62 ºC. Negative controls were produced by exposing the xylans to the same buffer and conditions but without the addition of xylanases. All reactions were performed in triplicate.

After the incubation, the enzymatic reactions were stopped by freezing for 2 h. After defrosting the samples, the content of each sample was diluted and analyzed.

### Analytical method

#### Ash and moisture content

The moisture and ash contents were determined by the method described by NREL [74, 75]. One gram of milled SCB was added to pre-dried and weighed crucibles and placed in an oven at 105 °C for 12 h. After transferring them to a desiccator and allowing them to cool at room temperature, the crucibles and samples were weighed. The moisture content was obtained by subtracting the weight of the crucible from the crucibles containing samples after and before oven-drying.

Ash content was measured by igniting the oven and drying samples at 575 °C to constant weight by using a muffle furnace equipped with a ramping program (Nabertherm, Germany). The percentage of ash content was determined by subtracting the weight of the crucible from the weight of the dried crucible containing ash, followed by dividing the ash with the weight of the oven dried sample.

#### Protein content

Protein content was estimated by quantifying the nitrogen content of bagasse using the Dumas method and an elemental analyser (Flash EA 1112, Thermo Fisher Scientific, USA). Twenty-five milligrams of the milled SCB were weighed into a tin container and subjected to combustion at 900–1000 °C in the presence of oxygen. The resulting nitrogen oxide was quantitatively converted to N_2_ in a reduction chamber containing copper at 650 °C, where other volatile products of combustion are either trapped or separated. Finally, the nitrogen gas was measured using a thermal conductivity detector. Aspartic acid was used for the calibration. Protein content was calculated with the nitrogen-to-protein conversion factor of 6.25.

#### Structural carbohydrates and lignin

The total structural carbohydrate and lignin content of the raw material, the treated SCB and the extracted xylans and cellulose were determined by a two-step sequential acid hydrolysis [76]. Three milliliters of sulphuric acid 72% (w/v) was added to 300 mg sample and was shaken at 200 rpm in the incubator at 30 °C for 60 min. Then, 84 mL of milli-Q water was added to the flask to dilute the acid to reach the concentration of 4% (w/v) and was autoclaved for 1 h at 121 °C. The autoclaved hydrolysis solutions were vacuum filtered through previously weighed filtering crucibles and all remaining solids were rinsed thoroughly. The crucibles were dried at 105 °C until constant weight to determine the acid-insoluble residue. The crucibles were placed in the muffle furnace at 575 °C to constant weight to measure acid-insoluble ash. Acid-insoluble lignin (Klasson lignin) was calculated by subtracting acid-soluble ash from acid-insoluble residue divided by the weight of the oven dried sample. Acid-soluble lignin was evaluated by measuring the absorbance of the vacuum filtered hydrolysis liquor at wavelength 240 nm using a UV/Vis spectrophotometer (Biochrom WPA Biowave II, UK).

To measure structural carbohydrates, the vacuum filtered hydrolysis liquor was neutralized by 0.1 M Ba(OH)_2_ and centrifuged (Sigma 3-16Pk) for 10 min at (3893 × g). Finally, after proper dilutions, the samples were filtered through 0.2 µm polypropylene filters and analyzed by a high-performance anion exchange chromatography system equipped with pulsed amperometric detector (HPAEC-PAD) (Thermo Fisher Scientific, Waltham, USA). A DionexCarboPac PA-20 analytical column and guard column (Thermo Fisher Scientific, Waltham, USA) were used to separate monosaccharides. Eluents were: (A) Ultrapure water (B) 2 mM NaOH and (C) 200 mM NaOH. Separation was done during 23 min running time using a mixture of A (62.5%) and B (37.5%) with an isocratic flow of 0.5 mL/min and after that the column was regenerated with C (100%) for 2 min at the same flow rate. Standards: arabinose, galactose, glucose, xylose and mannose were prepared with the concentration range of 10–60 µg/mL.

All the experiments were performed in duplicate and the mean value and standard deviation were calculated. Polymeric forms of carbohydrates (cellulose and hemicellulose xylan) were calculated based on a correction factor of 0.88 for C-5 sugars (xylose and arabinose) and 0.90 for C-6 sugars (glucose, galactose and mannose).

#### Acetyl content determination

The acetyl content was screened by using high-performance liquid chromatography (HPLC, Ultimate-3000 RSLC, Dionex) connected to an RI detector (Shodex, RI-101). One mL of the hydrolysis liquor collected after vacuum filtration, was acidified with 20 µL of sulfuric acid (20% v/v), and filtered through a 0.2 µm polypropylene filter. Separation of acetyl groups was done using the analytical column Aminex HPX-87H connected to a guard column (Biorad, Richmond, CA, USA). The temperature was set at 40 °C and the mobile phase consisted of 0.5 mM H_2_SO_4_ with a flow rate of 0.5 mL/min. Acetic acid was used as standard with the concentration range 25–200 μg/mL.

#### Uronic acids determination

Uronic acids determination and preparation of samples were like for acetyl content determination except that the temperature was set at 30 °C and the flow rate of the mobile phase was set at 0.3 mL/min. Glucuronic acid and galacturonic acid were used as standards with a concentration range of 50–1000 μg/mL.

#### Oligosaccharides determination

Oligosaccharide content was analyzed by HPAEC using a DionexCarboPac PA-200 analytical column (Thermo Fisher Scientific, Waltham, USA). Eluents were: (A) milliQ-water, (B) 400 mM sodium acetate in 200 mM NaOH, and (C) 200 mM NaOH. Oligosaccharides were separated by a linear gradient from zero to 45% (C) plus zero to 5% (B) in (A) at a flow rate of 0.5 mL/min for 20 min. Xylose, xylobiose, xylotriose, xylotetraose, xylopentaose and xylohexaose were used as standards with a concentration range of 5–80 μg/mL.

#### Molecular weight determination of purified extracted xylans

Molecular weight of the purified extracted xylans was estimated by size exclusion chromatography using a HPLC system (Agilent Technologies, USA) equipped with a Refractive-Index detector (ERC-7510, ERMA INC). A Sephacryl S-200 HR (1 × 10^3^–8 × 10^4^ Da) (HiPrep, GE healthcare life sciences) column (16 mm × 600 mm) was used to analyze the molecular weight of the xylans. Ultrapure water was used as an eluent at a flow rate of 0.3 mL/min for 500 min. Dextrans with molecular weights of 50, 80, 150, 270 and 670 kDa were used as standards to plot the standard curve.

#### Fourier transform infrared (FT-IR) spectroscopy

Fractions of purified xylan and cellulose, were freeze dried, and analyzed by Fourier transform infrared (FT-IR) spectroscopy by placing a portion of each sample in the diamond window of a FT-IR spectrometer (Thermo Scientific Nicolet iS5, USA) operating at 400–4000 cm^−1^ spectral range.

### Statistical analysis

Analysis of variance (ANOVA) was made by the data analysis tool from the Excel software (Microsoft) to evaluate if the differences between xylan extraction yields were significant. A 95% confidence level was used for the response variables, resulting in a significance level (*α*) of 0.05. A *p*-value lower than 0.05 for a term of the ANOVA table would therefore indicate statistical significance.

## Supplementary Information

**Additional file 1**: **Figure S1**. Validation plots for the PLE show the model fitting and prediction power (A and C) and coefficient plots (B and D) for cellulose and xylan. The coefficient plots show the direct and interaction effects of the investigated parameters on the solubility of cellulose and xylan. **Figure S2**. Response surface contour plots for lignin, hemicellulose and cellulose (%) in the remaining residue vs. the extraction factors; NaOH concentration (0-0.1 M), time (10-30 min) and temperature (50-150 °C). **Figure S3**. Size exclusion chromatography profiles of extracted xylans. Panel A. Dextran standards, Panel B. The profiles eluted after extraction(during 60 min) with 0.5M NaOH, 1M NaOH, 2M NaOH, respectively. **Figure S4**. HPAEC-PAD chromatogram of purified cellulose after acid hydrolysis.

## Data Availability

The data supporting the conclusions of this article are included within the article. Additional datasets from the current study are available from the corresponding author on reasonable request.

## References

[1] Zheng Y, Yu Y, Lin W, Jin Y, Yong Q, Huang C (2021). Enhancing the enzymatic digestibility of bamboo residues by biphasic phenoxyethanol-acid pretreatment. Bioresour Technol.

[2] Zheng L, Yu P, Zhang Y, Wang P, Yan W, Guo B, Huang C, Jiang Q (2021). Evaluating the bio-application of biomacromolecule of lignin-carbohydrate complexes (LCC) from wheat straw in bone metabolism via ROS scavenging. Int J Biol Macromol.

[3] Nordberg Karlsson E, Schmitz E, Linares-Pastén J, Adlercreutz P (2018). Endo-xylanases as tools for production of substituted xylooligosaccharides with prebiotic properties. Appl Microbiol Biotechnol.

[4] Pei W, Shang W, Liang C, Jiang X, Huang C, Yong Q (2020). Using lignin as the precursor to synthesize Fe3O4@lignin composite for preparing electromagnetic wave absorbing lignin-phenol-formaldehyde adhesive. Ind Crops Prod.

[5] Chen M-J, Shi Q-S (2015). Transforming sugarcane bagasse into bioplastics via homogeneous modification with phthalic anhydride in ionic liquid. ACS Sustain Chem Eng.

[6] Vargas Betancur GJ, Pereira N (2010). Sugar cane bagasse as feedstock for second generation ethanol production: part I: diluted acid pretreatment optimization. Electron J Biotechnol.

[7] Bian J, Peng F, Peng XP, Xu F, Sun RC, Kennedy JF (2012). Isolation of hemicelluloses from sugarcane bagasse at different temperatures: structure and properties. Carbohydr Polym.

[8] Pandey A, Soccol CR, Nigam P, Soccol VT (2000). Biotechnological potential of agro-industrial residues. I: sugarcane bagasse. Bioresour Technol.

[9] Wyman CE, Dale BE, Elander RT, Holtzapple M, Ladisch MR, Lee YY (2005). Comparative sugar recovery data from laboratory scale application of leading pretreatment technologies to corn stover. Bioresour Technol.

[10] Bezerra TL, Ragauskas AJ (2016). A review of sugarcane bagasse for second-generation bioethanol and biopower production. Biofuel Bioprod Biorefin.

[11] Cardona CA, Quintero JA, Paz IC (2010). Production of bioethanol from sugarcane bagasse: status and perspectives. Bioresour Technol.

[12] Brienzo M, Carvalho AFA, De Figueiredo FC, De Oliva Neto P, Riley GL (2016). Sugarcane bagasse hemicellulose properties, extraction technologies, and xylooligosaccharides production. Food waste: practices, management and challenges.

[13] Gil-Ramirez A, Salas-Veizaga DM, Grey C, Karlsson EN, Rodriguez-Meizoso I, Linares-Pastén JA (2018). Integrated process for sequential extraction of saponins, xylan and cellulose from quinoa stalks (Chenopodium quinoa Willd.). Ind Crops Prod.

[14] Naidu DS, Hlangothi SP, John MJ (2018). Bio-based products from xylan: a review. Carbohydr Polym.

[15] Canilha L, e Silva JBA, Felipe MG, Carvalho W (2003). Batch xylitol production from wheat straw hemicellulosic hydrolysate using *Candida guilliermondii* in a stirred tank reactor. Biotechnol Lett.

[16] Peng F, Peng P, Xu F, Sun R-C (2012). Fractional purification and bioconversion of hemicelluloses. Biotechnol Adv.

[17] Vázquez MJ, Alonso JL, Domínguez H, Parajó JC (2000). Xylooligosaccharides: manufacture and applications. Trends Food Sci Technol.

[18] Amorim C, Silvério SC, Prather KLJ, Rodrigues LR (2019). From lignocellulosic residues to market: production and commercial potential of xylooligosaccharides. Biotechnol Adv.

[19] Moure A, Gullón P, Domínguez H, Parajó JC (2006). Advances in the manufacture, purification and applications of xylo-oligosaccharides as food additives and nutraceuticals. Process Biochem.

[20] Falck P, Precha-atsawanan S, Grey C, Immerzeel P, Stålbrand H, Adlercreutz P, Nordberg KE (2013). XylooligosaccharidesfromHardwood and Cereal Xylans produced by a thermostable xylanase as Carbon Sources for *Lactobacillus brevis* and *Bifidobacterium adolescentis*. J Agric Food Chem.

[21] Turck D, Bresson JL, Burlingame B, Dean T, Fairweather-Tait S, Heinonen M, Hirsch-Ernst KI, Mangelsdorf I, McArdle HJ, Naska A, Neuhauser-Berthold M, Nowicka GZ, Pentieva K, Sanz Y, Siani A, Sjodin A, Stern M, Tome D, Vinceti M, Willatts P, Engel KH, Marchelli R, Poting A, Poulsen M, Schlatter JR, Turla E, van Loveren H, EFSA Panel on Dietetic Products, Nutrition and Allergies (NDA) (2018). Safety of xylo-oligosaccharides (XOS) as a novel food pursuant to Regulation (EU)2015/2283. EFSA J.

[22] Aachary AA, Gobinath D, Srinivasan K, Prapulla SG (2015). Protective effect of xylooligosaccharides from corncob on 1, 2-dimethylhydrazine induced colon cancer in rats. Bioact Carbohydr Diet Fibre.

[23] Kaur R, Uppal S, Sharma PJW, Valorization B (2019). Production of xylooligosaccharides from sugarcane bagasse and evaluation of their prebiotic potency in vitro. Waste Biomass Valoriz.

[24] Xue J-L, Zhao S, Liang R-M, Yin X, Jiang S-X, Su L-H, Yang Q, Duan C-J, Liu J-L, Feng J-X (2016). A biotechnological process efficiently co-produces two high value-added products, glucose and xylooligosaccharides, from sugarcane bagasse. Bioresour Technol.

[25] Berger K, Falck P, Linninge C, Nilsson U, Axling U, Grey C, Stålbrand H, Nordberg Karlsson E, Nyman M, Holm C, Adlercreutz P (2014). Cereal byproducts have prebiotic potential in mice fed a high-fat diet. J Agric Food Chem.

[26] Immerzeel P, Falck P, Galbe M, Adlercreutz P, Nordberg Karlsson E, Stålbrand H (2014). Extraction of water-soluble xylan from wheat bran and utilization of enzymatically produced xylooligosaccharides by Lactobacillus, Bifidobacterium and Weissella spp. LWT-Food Sci Technol.

[27] Valls C, Pastor FIJ, Vidal T, Roncero MB, Díaz P, Martínez J, Valenzuela SV (2018). Antioxidant activity of xylooligosaccharides produced from glucuronoxylan by Xyn10A and Xyn30D xylanases and eucalyptus autohydrolysates. Carbohydr Polym.

[28] Global Xylo-Oligosaccharide (XOS) market by manufacturers, regions, type and application, forecast to 2023. 2018. https://www.themarketreports.com/report/global-xylo-oligosaccharide-xos-market-by-manufacturers-regions-type-and-application-forecast-to-2023.

[29] Liu C-F, Ren J-L, Xu F, Liu J-J, Sun J-X, Sun R-C (2006). Isolation and characterization of cellulose obtained from ultrasonic irradiated sugarcane bagasse. J Agric Food Chem.

[30] Gordobil O, Egüés I, UrruzolA I, Labidi J (2014). Xylan–cellulose films: Improvement of hydrophobicity, thermal and mechanical properties. Carbohydr Polym.

[31] Klemm D, Heublein B, Fink HP, Bohn A (2005). Cellulose: fascinating biopolymer and sustainable raw material. Angew Chem Int Ed.

[32] Pandey JK, Nakagaito AN, Takagi H (2013). Fabrication and applications of cellulose nanoparticle-based polymer composites. Polym Eng Sci.

[33] Huang C, Dong H, Zhang Z (2020). Procuring the nano-scale lignin in prehydrolyzate as ingredient to prepare cellulose nanofibril composite film with multiple functions. Cellulose.

[34] Xiang Z, Jin X, Huang C (2020). Water cast film formability of sugarcane bagasse xylans favored by side groups. Cellulose.

[35] Sun JX, Sun XF, Sun RC, Su YQ (2004). Fractional extraction and structural characterization of sugarcane bagasse hemicelluloses. Carbohydr Polym.

[36] Kumar AK, Sharma S (2017). Recent updates on different methods of pretreatment of lignocellulosic feedstocks: a review. Bioresour Bioprocess.

[37] Lee YY, Iyer P, Torget RW, Tsao GT (1999). Dilute-acid hydrolysis of lignocellulosic biomass. Recent progress in bioconversion of lignocellulosics. Advances in biochemical engineering/biotechnology.

[38] Saska M, Ozer E (1995). Aqueous extraction of sugarcane bagasse hemicellulose and production of xylose syrup. Biotechnol Bioeng.

[39] Shimizu K, Sudo K, Ono H, Ishihara M, Fujii T, Hishiyama S (1998). Integrated process for total utilization of wood components by steam-explosion pretreatment. Biomass Bioenergy.

[40] Jayapal N, Samanta AK, Kolte AP, Senani S, Sridhar M, Suresh KP, Sampath KT (2013). Value addition to sugarcane bagasse: Xylan extraction and its process optimization for xylooligosaccharides production. Ind Crops Prod.

[41] van der Pol E, Bakker R, van Zeeland A, Garcia DS, Punt A, Eggink G (2015). Analysis of by-product formation and sugar monomerization in sugarcane bagasse pretreated at pilot plant scale: differences between autohydrolysis, alkaline and acid pretreatment. Bioresour Technol.

[42] Boonchuay P, Techapun C, Leksawasdi N, Seesuriyachan P, Hanmoungjai P, Watanabe M, Takenaka S, Chaiyaso T (2018). An integrated process for xylooligosaccharide and bioethanol production from corncob. Bioresour Technol.

[43] Chen H, Liu J, Chang X, Chen D, Xue Y, Liu P, Lin H, Han S (2017). A review on the pretreatment of lignocellulose for high-value chemicals. Fuel Process Technol.

[44] Huang H-J, Ramaswamy S, Tschirner UW, Ramarao BV (2008). A review of separation technologies in current and future biorefineries. Sep Purif Technol.

[45] Peng F, Ren J-L, Xu F, Bian J, Peng P, Sun R-C (2009). Comparative study of hemicelluloses obtained by graded ethanol precipitation from sugarcane bagasse. J Agric Food Chem.

[46] Sporck D, Reinoso FAM, Rencoret J, Gutiérrez A, del Rio JC, Ferraz A, Milagres AMF (2017). Xylan extraction from pretreated sugarcane bagasse using alkaline and enzymatic approaches. Biotechnol Biofuels.

[47] Samanta AK, Senani S, Kolte AP, Sridhar M, Sampath KT, Jayapal N, Devi A (2012). Production and in vitro evaluation of xylooligosaccharides generated from corn cobs. Food Bioprod Process.

[48] Linares-Pastén JA, Aronsson A, Nordberg Karlsson E (2018). Structural considerations on the use of endo-xylanases for the production of prebiotic xylooligosaccharides from biomass. Curr Protein Pept Sci.

[49] Bian J, Peng F, Peng X-P, Peng P, Xu F, Run-Cang Sun R-C (2013). Structural features and antioxidant activity of xylooligosaccharides enzymatically produced from sugarcane bagasse. Bioresour Technol.

[50] Brienzo M, Carvalho W, Milagres AMF (2010). Xylooligosaccharides production from alkali-pretreated sugarcane bagasse using xylanases from *Thermoascus aurantiacus*. Appl Biochem Biotechnol.

[51] de Moraes Rocha GJ, Nascimento VM, Gonçalves AR, Silva VFN, Martín C (2015). Influence of mixed sugarcane bagasse samples evaluated by elemental and physical–chemical composition. Ind Crops Prod.

[52] Humpert D, Ebrahimi M, Czermak P (2016). Membrane technology for the recovery of lignin: a review. Membranes.

[53] Mustafa A, Turner C (2011). Pressurized liquid extraction as a green approach in food and herbal plants extraction: a review. Anal Chim Acta.

[54] Kilpeläinen P, Leppänen K, Spetz P, Kitunen V, Ilvesniemi H, Pranovich A, Willför, (2012). Biorefinery. Pressurised hot water extraction of acetylated xylan from birch sawdust. Nord Pulp Pap Res J.

[55] Ebringerová A (2005). Structural diversity and application potential of hemicelluloses. Macromol Symp.

[56] Benjamin Y, Cheng H, Görgens JF (2014). Optimization of dilute sulfuric acid pretreatment to maximize combined sugar yield from sugarcane bagasse for ethanol production. Appl Biochem Biotechnol.

[57] Brienzo M, Siqueira A, Milagres AMF (2009). Search for optimum conditions of sugarcane bagasse hemicellulose extraction. Biochem Eng J.

[58] Costa THF, Vega-Sánchez ME, Milagres AMF, Scheller HV, Ferraz A (2016). Tissue-specific distribution of hemicelluloses in six different sugarcane hybrids as related to cell wall recalcitrance. Biotechnol Biofuels.

[59] Morais de Carvalho D, Martínez-Abad A, Evtuguin DV, Colodette JL, Lindström ME, Vilaplana F, Sevastyanova O (2017). Isolation and characterization of acetylated glucuronoarabinoxylan from sugarcane bagasse and straw. Carbohydr Polym.

[60] Koegelenberg D, Chimphango AFA (2017). Effects of wheat-bran arabinoxylan as partial flour replacer on bread properties. Food Chem.

[61] Liu K, Zhang J, Li H, Xu J (2016). Effect of alkali concentration and temperature on extraction yield and quality of xylan from corn stover. Trans Chin Soc Agric Eng.

[62] Salas-VeizagA DM, Villagomez R, Linares-Pastén JA, Carrasco C, Álvarez MT, Adlercreutz P, Nordberg Karlsson E (2017). Extraction of glucuronoarabinoxylan from quinoa stalks (*Chenopodium quinoa* Willd.) and evaluation of xylooligosaccharides produced by GH10 and GH11 xylanases. J Agric Food Chem.

[63] Capek P, Matulová M (2013). An arabino(glucurono)xylan isolated from immunomodulatory active hemicellulose fraction of *Salvia officinalis L*. Int J Biol Macromol.

[64] Hospodarova V, Singovszka E, Stevulova N. Characterization of cellulosic fibers by FTIR spectroscopy for their further implementation to building materials. Am J Anal Chem 2018;9: 303–310. www.scirp.org/journal/ajac

[65] Rosa MF, Medeiros ES, Malmonge JA, Gregorski KS, Wood DF, Mattoso LHC, Glenn G, Orts WJ, Imam SH (2010). Cellulose nanowhiskers from coconut husk fibers: effect of preparation conditions on their thermal and morphological behavior. Carbohydr Polym.

[66] Popescu M-C, Popescu C-M, Lisa G, Sakata Y (2011). Evaluation of morphological and chemical aspects of different wood species by spectroscopy and thermal methods. J Mol Struct.

[67] Rodrigues J, Faix O, Pereira H (1998). Determination of lignin content of *Eucalyptus globulus* wood using FTIR spectroscopy. Holzforschung.

[68] Kiran EU, Akpinar O, Bakir U (2013). Improvement of enzymatic xylooligosaccharides production by the co-utilization of xylans from different origins. Food bioprod process.

[69] Gullón P, Moura P, Esteves MAP, Girio FM, Domínguez H, Parajó JC (2008). Assessment on the fermentability of xylooligosaccharides from rice husks by probiotic bacteria. J Agric Food Chem.

[70] Zhang W, You Y, Lei F, Li P, Jiang J (2018). Acetyl-assisted autohydrolysis of sugarcane bagasse for the production of xylo-oligosaccharides without additional chemicals. Bioresour Technol.

[71] Dai L, Huang T, Jiang K (2021). A novel recyclable furoic acid-assisted pretreatment for sugarcane bagasse biorefinery in co-production of xylooligosaccharides and glucose. Biotechnol Biofuels.

[72] Lin W, Chen D, Yong Q, Huang C, Huang S (2019). Improving enzymatic hydrolysis of acid-pretreated bamboo residues using amphiphilic surfactant derived from dehydroabietic acid. Bioresour Technol.

[73] Bian J, Peng F, Peng P, Xu F, Sun R-C (2010). Isolation and fractionation of hemicelluloses by graded ethanol precipitation from Caragana korshinskii. Carbohydr Res.

[74] Sluiter A, Hames B, Hyman D, Payne C, Ruiz R, Scarlata C, Sluiter J, Templeton D, Wolfe J. Determination of total solids in biomass and total dissolved solids in liquid process samples. Technical Report. NREL/TP-510–42621; 2008a

[75] Sluiter A, Hames B, Ruiz R, Scarlata C, Sluiter J, Templeton D, Crocker D. Determination of structural carbohydrates and lignin in biomass. Technical Report NREL/TP-510-42618; 2008b.

[76] Sluiter A, Hames B, Ruiz R, Scarlata C, Sluiter J, Templeton D. Determination of ash in biomass. Technical ReportNREL/TP-510-42622; 2008c

