# Supplementary material for: Extraction of sugarcane bagasse arabinoxylan, integrated with enzymatic production of xylo-oligosaccharides and separation of cellulose
Source: Biotechnol Biofuels. 2021 Jul 3;14:153. doi: 10.1186/s13068-021-01993-z (PMC8254973; doi:10.1186/s13068-021-01993-z)

**Extraction of sugarcane bagasse arabinoxylan, integrated with enzymatic production of xylo-oligosaccharides and separation of cellulose**

Leila Khaleghipour^a,b^, Javier A. Linares-Pastén^a^, Hamid Rashedi^b^, Seyed Omid Ranaei Siadat^c^, Andrius Jasilionis^a^, Said Al-Hamimi^d^, Roya R. R. Sardari^a^, Eva Nordberg Karlsson^a^

^a^ Division of Biotechnology, Department of Chemistry, Lund University, P. O. Box 124, 22100 Lund, Sweden. ^b^ Biotechnology Group, School of Chemical Engineering, College of Engineering, University of Tehran, Tehran, Iran. ^c^ Protein Research Center, Shahid Beheshti University, G. C., Tehran, Iran. **^d^** Center for Analysis and Synthesis, Department of Chemistry, Lund University, P. O. Box 124, 22100 Lund, Sweden

*Corresponding Authors:* [*eva.nordberg_karlsson@biotek.lu.se*](mailto:eva.nordberg_karlsson@biotek.lu.se)*,* [*hrashedi@ut.ac.*ir](mailto:hrashedi@ut.ac.ir)

*_____________________________________________________________________________________________*

**Supplementary Figure S1.** Validation plots for the PLE show the model fitting and prediction power (A and C) and coefficient plots (B and D) for cellulose and xylan. The coefficient plots show the direct and interaction effects of the investigated parameters on the solubility of cellulose and xylan.


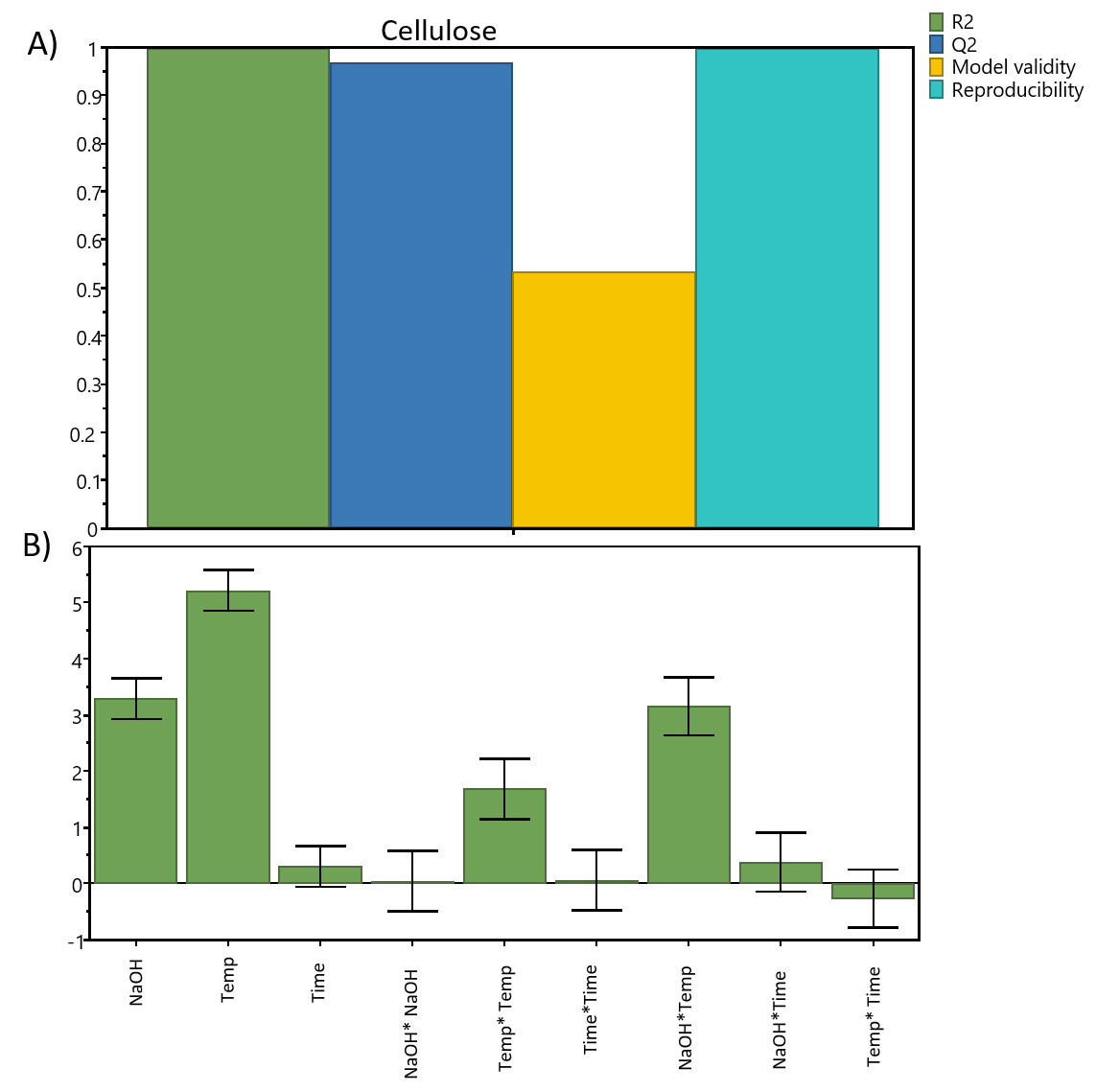


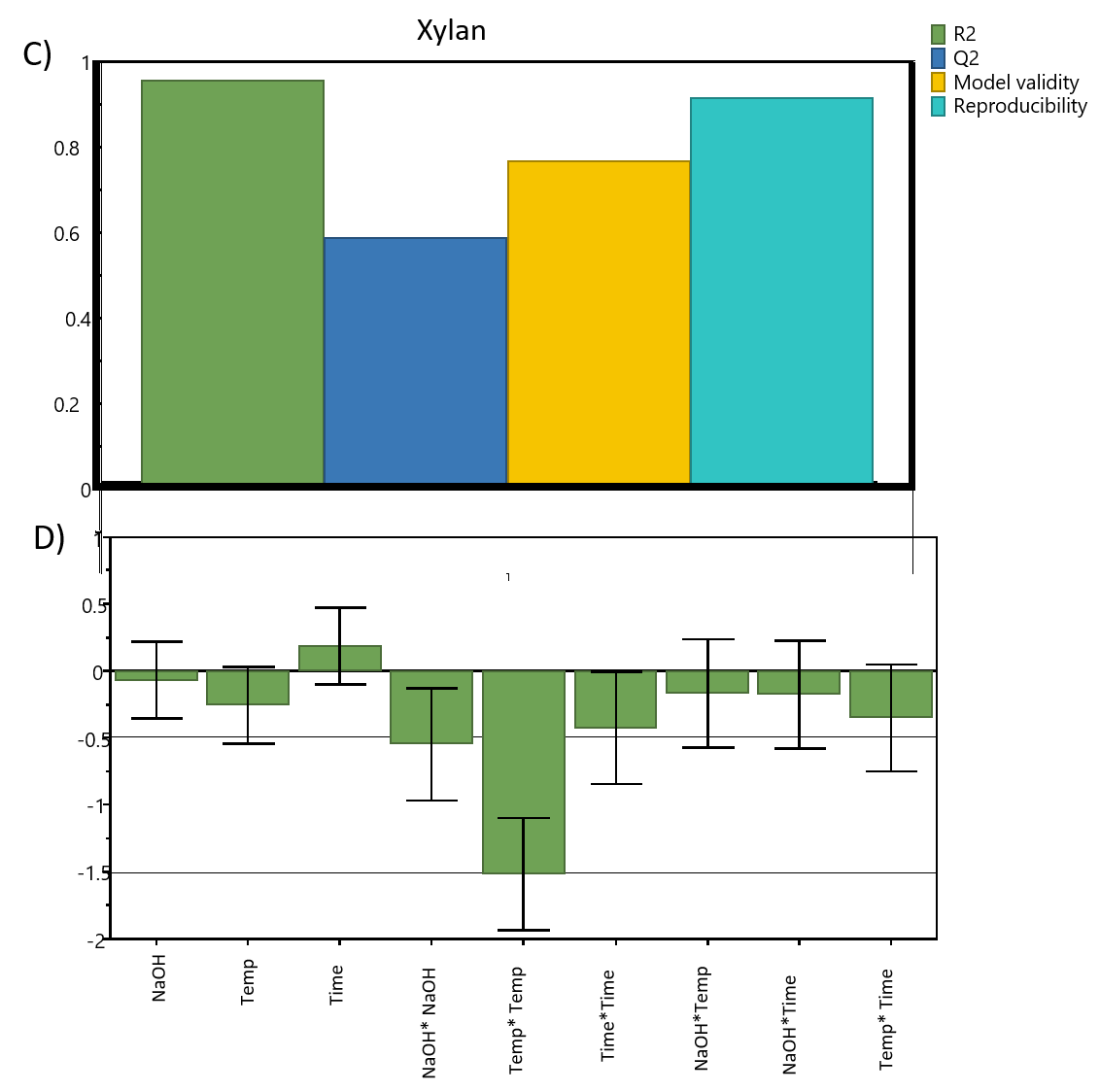


**Supplementary Figure S2.** Response surface contour plots for lignin, hemicellulose and cellulose (%) in the remaining residue vs. the extraction factors; NaOH concentration (0-0.1 M), time (10-30 min) and temperature (50-150 °C).


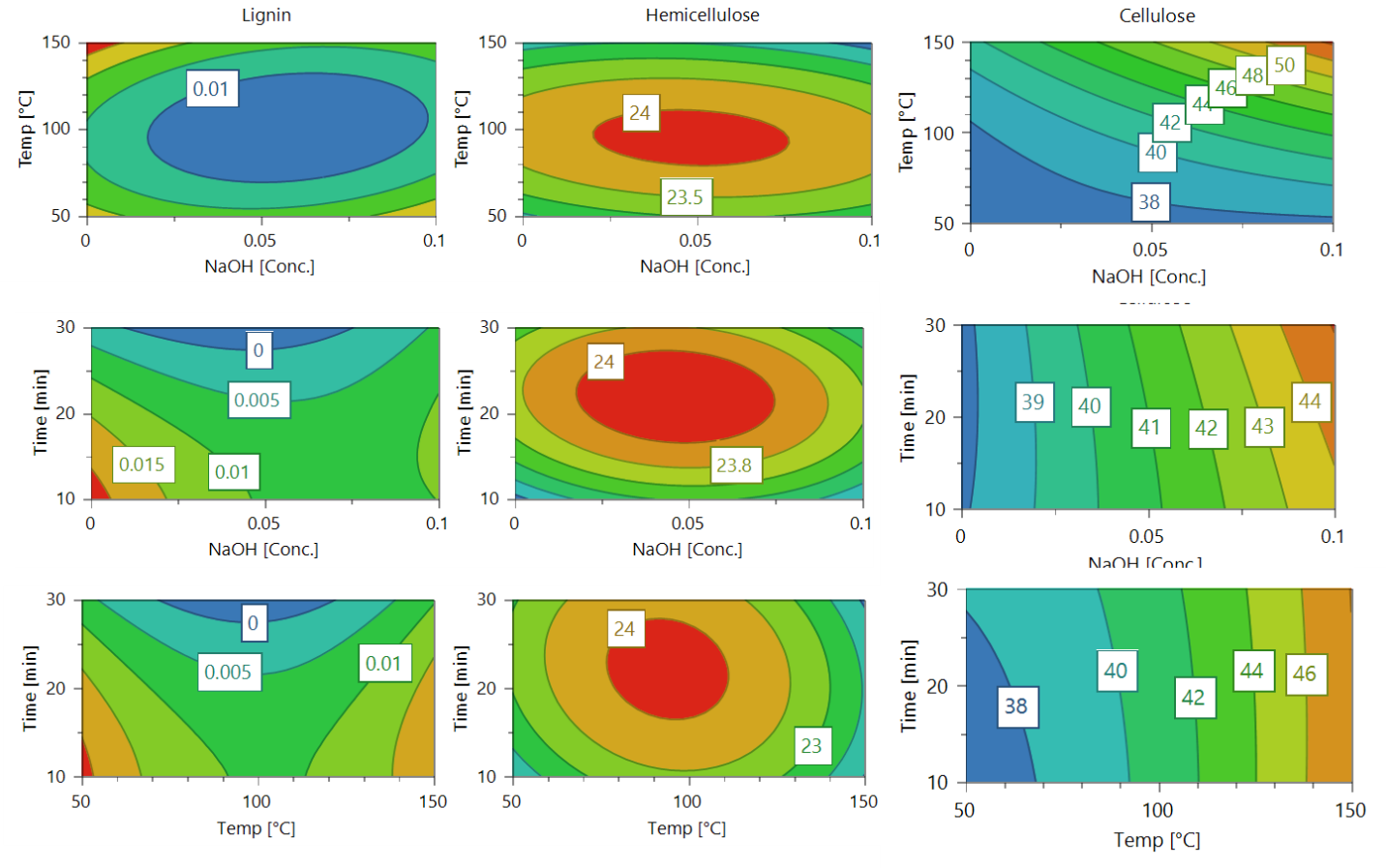


**Supplementary Figure S3.** Size exclusion chromatography profiles of extracted xylans. Panel A. Dextran standards, Panel B. The profiles eluted after extraction (during 60 min) with 0.5M NaOH, 1M NaOH, 2M NaOH, respectively.


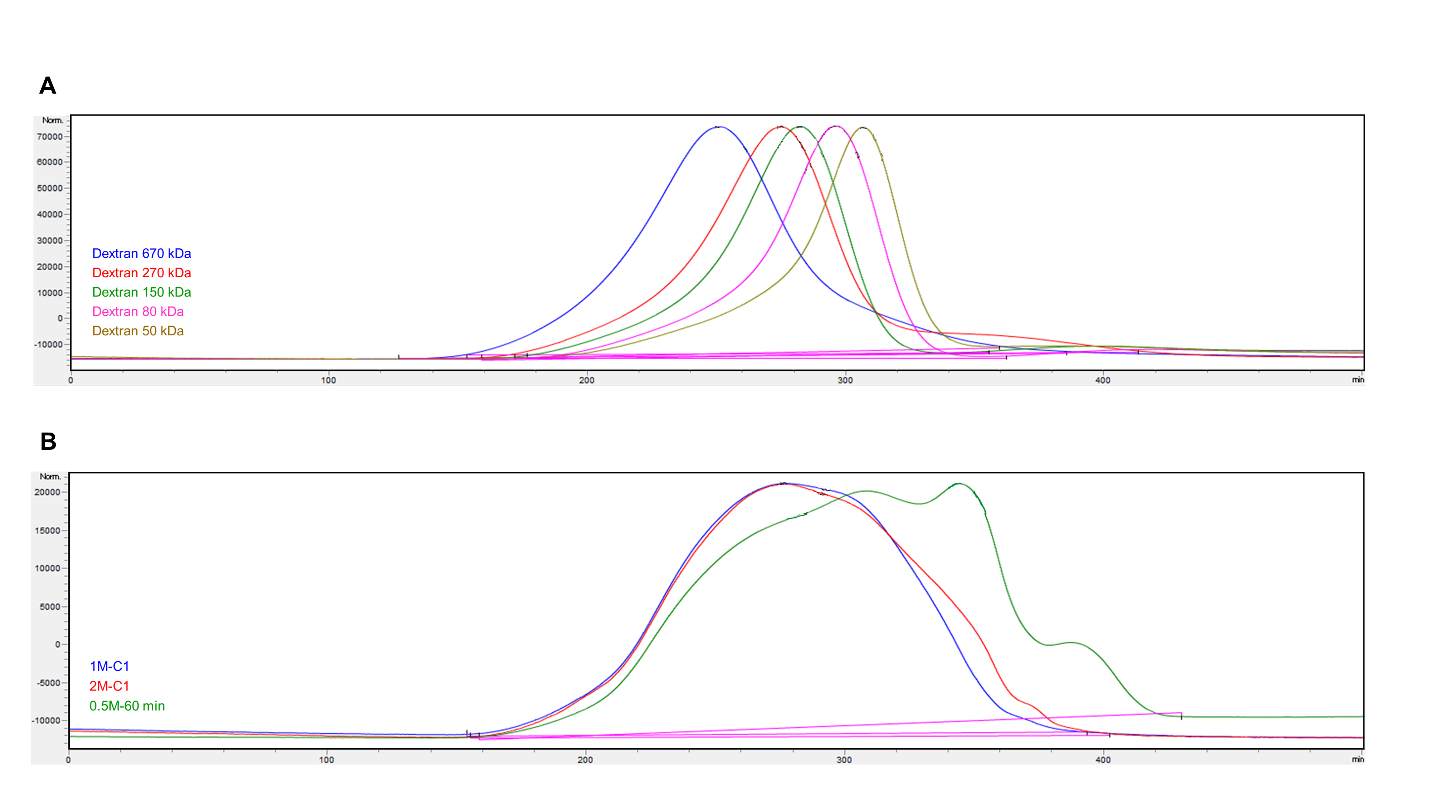


**Supplementary Figure S4.** HPAEC-PAD chromatogram of purified cellulose after acid hydrolysis.


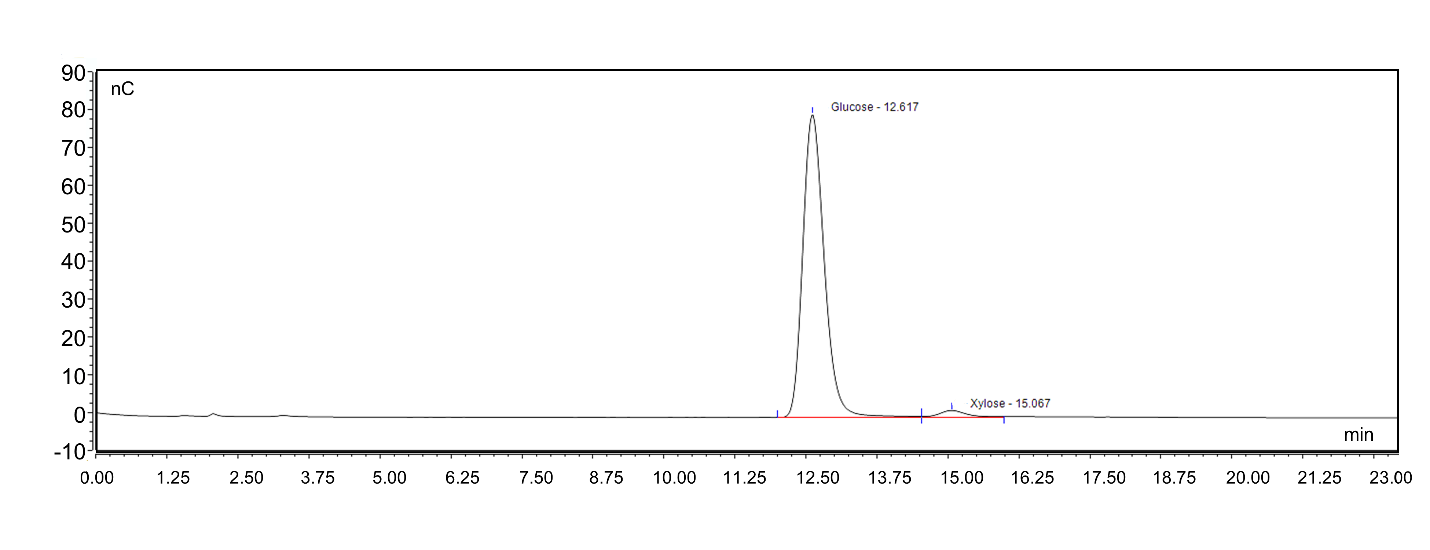

Supplement: Supplementary file 1 — Additional file 1: Figure S1. Validation plots for the PLE show the model fitting and prediction power (A and C) and coefficient plots (B and D) for cellulose and xylan. The coefficient plots show the direct and interaction effects of the investigated parameters on the solubility of cellulose and xylan. Figure S2. Response surface contour plots for lignin, hemicellulose and cellulose (%) in the remaining residue vs. the extraction factors; NaOH concentration (0-0.1 M), time (10-30 min) and temperature (50-150 °C). Figure S3. Size exclusion chromatography profiles of extracted xylans. Panel A. Dextran standards, Panel B. The profiles eluted after extraction(during 60 min) with 0.5M NaOH, 1M NaOH, 2M NaOH, respectively. Figure S4. HPAEC-PAD chromatogram of purified cellulose after acid hydrolysis. [file 13068_2021_1993_MOESM1_ESM.docx]
